# Biological mechanisms of resilience to tau pathology in Alzheimer’s disease

**DOI:** 10.1186/s13195-024-01591-9

**Published:** 2024-10-12

**Authors:** Anna L. Svenningsson, Diana I. Bocancea, Erik Stomrud, Anita van Loenhoud, Frederik Barkhof, Niklas Mattsson-Carlgren, Sebastian Palmqvist, Oskar Hansson, Rik Ossenkoppele

**Affiliations:** 1 Clinical Memory Research Unit, Department of Clinical Sciences Malmö, Faculty of Medicine, Lund University, 211 46 Lund, Sweden; 2 Memory Clinic, Skåne University Hospital, 214 28 Malmö, Sweden; 3 Alzheimer Center Amsterdam, Neurology, Vrije Universiteit Amsterdam, Amsterdam UMC Location VUmc, 1081 HZ Amsterdam, The Netherlands; 4 Amsterdam Neuroscience, Neurodegeneration, 1081 Amsterdam, The Netherlands; 5 Department of Radiology and Nuclear Medicine, Vrije Universiteit Amsterdam, Amsterdam UMC, 1081 HV Amsterdam, The Netherlands; 6 Queen Square Institute of Neurology and Center for Medical Image Computing, University College London, London, WC1N 3BG UK; 7 Department of Neurology, Skåne University Hospital, 211 84 Lund, Sweden; 8 Wallenberg Center for Molecular Medicine, Lund University, Lund, Sweden

**Keywords:** Alzheimer’s disease, Tau, Brain resilience, Cognitive resilience

## Abstract

**Background:**

In Alzheimer’s disease (AD), the associations between tau pathology and brain atrophy and cognitive decline are well established, but imperfect. We investigate whether cerebrospinal fluid (CSF) biomarkers of biological processes (vascular, synaptic, and axonal integrity, neuroinflammation, neurotrophic factors) explain the disconnection between tau pathology and brain atrophy (brain resilience), and tau pathology and cognitive decline (cognitive resilience).

**Methods:**

We included 428 amyloid positive participants (134 cognitively unimpaired (CU), 128 with mild cognitive impairment (MCI), 166 with AD dementia) from the BioFINDER-2 study. At baseline, participants underwent tau positron emission tomography (tau-PET), magnetic resonance imaging (MRI), cognitive testing, and lumbar puncture. Longitudinal data were available for MRI (mean (standard deviation) follow-up 26.4 (10.7) months) and cognition (25.2 (11.4) months). We analysed 18 pre-selected CSF proteins, reflecting vascular, synaptic, and axonal integrity, neuroinflammation, and neurotrophic factors. Stratifying by cognitive status, we performed linear mixed-effects models with cortical thickness (brain resilience) and global cognition (cognitive resilience) as dependent variables to assess whether the CSF biomarkers interacted with tau-PET levels in its effect on cortical atrophy and cognitive decline.

**Results:**

Regarding brain resilience, interaction effects were observed in AD dementia, with vascular integrity biomarkers (VEGF-A (β_interaction_ = -0.009, p_FDR_ = 0.047) and VEGF-B (β_interaction_ = -0.010, p_FDR_ = 0.037)) negatively moderating the association between tau-PET signal and atrophy. In MCI, higher NfL levels were associated with more longitudinal cortical atrophy (β = -0.109, p_FDR_ = 0.033) and lower baseline cortical thickness (β = -0.708, p_FDR_ = 0.033) controlling for tau-PET signal. Cognitive resilience analyses in CU revealed interactions with tau-PET signal for inflammatory (GFAP, IL-15; β_interaction_ -0.073–-0.069, p_FDR_ 0.001–0.045), vascular (VEGF-A, VEGF-D, PGF; β_interaction_ -0.099–-0.063, p_FDR_ < 0.001–0.046), synaptic (14–3-3ζ/δ; β_interaction_ = -0.092, p_FDR_ = 0.041), axonal (NfL; β_interaction_ = -0.079, p_FDR_ < 0.001), and neurotrophic (NGF; β_interaction_ = 0.091, p_FDR_ < 0.001) biomarkers. In MCI higher NfL levels (β_main_ = -0.690, p_FDR_ = 0.025) were associated with faster cognitive decline independent of tau-PET signal.

**Conclusions:**

Biomarkers of co-existing pathological processes, in particular vascular pathology and axonal degeneration, interact with levels of tau pathology on its association with the downstream effects of AD pathology (i.e. brain atrophy and cognitive decline). This indicates that vascular pathology and axonal degeneration could impact brain and cognitive resilience.

**Supplementary Information:**

The online version contains supplementary material available at 10.1186/s13195-024-01591-9.

## Background

Amyloid-β (Aβ) plaques and tau neurofibrillary tangles are two neuropathological hallmarks of Alzheimer’s disease (AD) where previous studies have indicated that tau pathology is more strongly associated with both concurrent and longitudinal neurodegeneration as well as cognitive decline [1–3]. However, these associations are imperfect and show considerable interindividual differences [4] with comparable burden of Aβ and tau pathology resulting in variable levels of cognitive impairment or neurodegeneration [5, 6]. Resilience is a concept trying to explain the observation that some people have better than expected brain structure (brain resilience [BR]) or cognitive performance (cognitive resilience [CR]), given either the level of a measurable pathology or the presence of a risk factor for disease [7, 8]. In the context of AD, variables such as intracranial volume for BR [7, 9] and educational attainment or intelligence quotient (IQ) for CR [10–13] have been identified and used as markers of resilience.

The aforementioned variables, however, are proxies that only indirectly measure resilience and only little is known about possible underlying biological mechanisms that provide BR and/or CR. Functional imaging studies in subjects without dementia using resting state functional magnetic resonance imaging (MRI) [14–16] have shown associations between higher amyloid burden and increased connectivity and activation, and others have shown associations between higher amyloid burden and increased metabolism using fluorodeoxyglucose-positron emission tomography (FDG-PET) [17, 18], which could indicate a compensatory mechanism early in the AD trajectory. One study examining cerebrospinal fluid (CSF) proteins in the context of resilience showed that the disconnection between AD biomarker levels and severity of symptoms could, to a large extent, be explained by vascular, lipid-metabolic and immune-related biomarkers in CSF, with for example higher CSF levels of vascular endothelial growth factor (VEGF) being associated with better cognitive performance and explaining some of the variance in cognition not explained by core AD biomarkers [19]. In addition, there are genetic studies that have investigated resilience in AD, showing putative roles for genes associated with longevity, vascular risk, metabolism, and mental health [20, 21].

However, few studies have investigated the longitudinal associations of biomarkers of possible biological underpinnings of resilience with atrophy and cognitive decline, and especially whether these associations differ depending on the level of tau pathology. Therefore, in this longitudinal study of participants across the AD spectrum, we investigated whether a set of pre-selected CSF biomarkers, that reflect different molecular processes, can explain some of the observed interindividual differences in the association between the amount of tau pathology and atrophy (brain resilience) or cognitive decline (cognitive resilience) over time.

## Methods

### Participants

The BioFINDER-2 study (NCT03174938) is a longitudinal cohort study investigating neurodegenerative disorders such as Alzheimer’s disease and parkinsonian disorders, including patients with mild cognitive impairment (MCI) or dementia as well as cognitively unimpaired (CU) volunteers. The participants undergo repeated clinical evaluations, cognitive testing, [^18^F]RO948 PET (tau PET), MRI, and lumbar punctures. For this study, participants from BioFINDER-2 were included if they were 50 years or older, amyloid positive at baseline as determined by the CSF Aβ42/40 ratio and had available baseline tau PET and CSF. From these, all participants who were cognitively unimpaired were included. Participants with dementia were included if they fulfilled the DSM-5 criteria of Alzheimer’s disease with major neurocognitive disorder. Due to the small sample size of the MCI group, participants with MCI were included if they fulfilled the DSM-5 criteria of Alzheimer’s disease with minor neurocognitive disorder or if their diagnosis was not determined, i.e. they were amyloid positive and no other neurological condition explained their cognitive symptoms but they did not fulfil criteria for AD. For the brain resilience analyses, participants with at least two MRI scans were included and for the cognitive resilience analyses, participants with at least two cognitive assessment visits were included. Participants who did not have CSF data from less than 18 months before or after the tau PET or did not have an MRI less than 12 months before or after the tau PET were excluded. The study was approved by the ethics committee at Lund University and the participants gave their written informed consent.

[^18^F]RO948 PET acquisition and processing.

Participants underwent [^18^F]RO948 PET scanning on a digital GE Discovery MI scanner 70–90 min following injection. Standardized uptake value ratios (SUVRs) were created using the inferior cerebellum as reference region [22]. Mean regional SUVRs were extracted using the cross-sectional FreeSurfer parcellation (version 6.0; http://surfer.nmr.mgh.harvard.edu/) of T1-weighted MRI scans. For main analyses, we calculated a temporal meta region-of-interest (ROI) from the entorhinal, parahippocampal, fusiform, inferior temporal, and middle temporal cortices and amygdala volume, referred to as temporal meta-ROI [23]. For secondary analyses, we used whole brain uptake, averaging SUVRs from 68 FreeSurfer cortical regions from both hemispheres [24].

### MRI acquisition and processing

T1-weighted MRI images were acquired on a 3 Tesla MAGNETOM Prisma scanner. The longitudinal pipeline [25] from FreeSurfer version 6.0 was used to extract cortical thickness measures. For BR analyses, we used the average thickness of the bilateral entorhinal, inferior temporal, middle temporal, and fusiform cortices [26], referred to as AD signature cortical thickness, as the primary outcome. For secondary analyses, we used the mean area weighted cortical thickness across all 68 cortical regions of interest from the Desikan-Killiany atlas [27], referred to as whole brain cortical thickness.

### Cognition

For CR analyses, global cognition was used as the primary outcome. We used Mini Mental State Examination (MMSE) [28] and a modified version of the Preclinical Alzheimer Cognitive Composite (mPACC5) [29], including averaged z-scores of MMSE, trailmaking test A (TMTA; multiplied by -1 to make a higher value represent better cognition), animal fluency, and the Alzheimer Disease Assessment Scale-Cognitive Subscale (ADAS-Cog) delayed recall weighted double since the original PACC5 includes two memory tests [30]. We used mPACC5 as primary outcome in CU since PACC5 is sensitive for cognitive decline in this group [30] and MMSE as primary outcome in MCI and AD dementia participants. For secondary analyses we investigated two domain specific tests, i.e. the average of the three attempts in ADAS-Cog immediate recall for memory [31] and TMTA for cognitive speed [32].

### Cerebrospinal fluid biomarkers

Results from the CSF collected closest to the tau PET for each participant were used. Lumbar CSF was collected and stored in -80° pending analysis. Levels of Aβ42 and Aβ40 were measured using Elecsys immunoassays [33]. We used the predefined Aβ42/40 ratio < 0.080 as the cut-off for determining amyloid positivity [34].

Based on previous literature in the field of AD, we investigate 18 pre-selected CSF proteins from different molecular pathways reflecting neuroinflammation, vascular integrity, synaptic integrity, axonal integrity, and neurotrophic factors. We used glial fibrillary acidic protein (GFAP) [35, 36], triggering receptor expressed on myeloid cells 2 (TREM2) [37, 38], progranulin (GRN) [39], intercellular adhesion molecule 1 (ICAM-1) [40], interleukin 15 (IL-15) [40], and chitinase-3-like protein 1 (YKL-40) [40] as neuroinflammatory markers and vascular endothelial growth factors (VEGF-A-D and placental growth factor (PGF)) as markers of vascular integrity [41, 42]. Neuronal pentraxin 2 (NPTX2) [43–45], neurogranin (NRGN) [46], synaptotagmin 1 (SYT1) [47], synaptic vesicle glycoprotein 2A (SV2A) [48], and 14–3-3 ζ/δ [44] were included as markers of synaptic integrity, and neurofilament light (NfL) as a marker of axonal integrity [49–51]. We also included nerve growth factor (NGF) as a neurotrophic factor [52].

Levels of all CSF proteins except for 14–3-3 ζ/δ were quantified using the validated, highly sensitive and specific multiplex immunoassay developed by Olink Proteomics (Uppsala, Sweden) [53]. Briefly, a unique deoxyribonucleic acid (DNA) sequence forms through hybridization of two complementary oligonucleotides, attached to antibodies that bind to the specific proteins, when these oligonucleotides are in proximity to each other in the presence of DNA polymerase. This DNA sequence is then detected using real-time quantitative polymerase chain reaction (qPCR). The cycled threshold (Ct) values rendered from the qPCR are then translated using a series of calculations to Normalized Protein eXpression (NPX) values, a log2-scale quantity for relative quantification of protein abundance (i.e. a 1 NPX difference represents a doubling of protein concentration). Samples have gone through appropriate internal and external quality controls from the manufacturer. Measures were excluded from our analyses if the quality control generated a warning. To adjust our models for possible effects of individual differences in CSF dynamics, we calculated the “mean NPX” variable as the z-scored average of all highly detected proteins in the assay, defined as proteins with less than 10% of the samples below the limit of detection (*n* = 1157).

Levels of CSF 14–3-3 ζ/δ were measured using a mass spectrometry-based panel of synaptic biomarkers [54]. The values were log2 transformed to put them on a similar scale as the Olink variables.

The number of missing values varied between the different CSF biomarkers, see Supplementary Table 1 for information on missing values per clinical group. The distributions of the CSF biomarkers are shown in Supplementary Fig. 1.

### Statistics

For interpretation purposes, continuous variables were z-scored using the means and standard deviations from a sample of amyloid negative cognitively unimpaired participants from BioFINDER-2 (*n* = 468, not included in the current analyses where we only focused on amyloid positive individuals). Analyses were performed within CU, MCI, and AD dementia subjects separately because we hypothesized that different proteins could be significant in the different groups and/or the direction of the interactions could vary between different clinical stages. All statistical analyses and data processing were conducted in R version 4.2.1.

### Brain resilience (BR) and cognitive resilience (CR) definitions

We define brain resilience (BR) as better than expected brain structure (i.e. cortical thickness) and cognitive resilience (CR) as better than expected cognitive performance [7, 8] given the level of tau pathology at baseline, measured using tau PET. We test for interaction effects of different CSF biomarkers on the association between tau pathology and atrophy (to assess BR) and cognitive decline (to assess CR), as well as independent main effects of these CSF biomarkers on atrophy and cognitive decline controlling for tau PET levels. A significant interaction effect indicates that the association between tau pathology and atrophy or cognitive decline differs depending on the level of the CSF biomarker. A significant main effect of the studied CSF biomarker when controlling for tau PET levels indicates that the rate of atrophy or cognitive decline differs depending on the CSF concentration of this biomarker independent of the level of tau pathology. Both instances can be interpreted as having lower or higher CSF levels of the studied biomarker results in better or worse brain structure or cognitive performance given the level of tau pathology, i.e. contributing to, or depleting, brain or cognitive resilience.

### Bivariate models

We used linear mixed-effects models with longitudinal cortical thickness measures as outcome in BR analyses and longitudinal cognitive tests as outcome in CR analyses. Baseline levels of tau (temporal meta-ROI for main analyses), time since tau PET, and the interaction term between time and tau PET were included in all analyses, covarying for age, sex, and mean NPX, as well as their interactions with time (as the longitudinal accumulation of tau pathology and time to disease progression can differ between males and females or participants of different ages [55, 56]). Separate analyses were performed for each CSF biomarker. Two models were tested. First, we tested for interaction effects for each CSF biomarker with tau PET signal on longitudinal atrophy (BR analyses) or cognitive decline (CR analyses), i.e. if the term biomarker*tau*time was significant. Second, if no significant interaction was found, we tested for independent effects of the biomarker on the longitudinal outcome measure or the outcome measure at baseline (i.e. at time = 0) when controlling for tau, i.e. if the term biomarker*time was significant controlling for tau signal and other covariates. For biomarkers where the interaction term with tau PET levels was significant, we present both conditional (i.e. results from models including the interaction term) and independent (i.e. results from models without the interaction term) cross-sectional and longitudinal main effects. The interpretation of the coefficient of the conditional main effects is that it is the effect each CSF biomarker has on the outcome when the tau variable is 0 (i.e. taking out the interaction term with tau), which in this case (because of our standardizing procedure) reflects the mean tau level in a group of amyloid negative CU. For biomarkers where the interaction term with tau PET levels was not significant, we only present independent (not conditional) cross-sectional and longitudinal main effects. CR analyses were run with random intercept and slope (apart from secondary analyses using TMTA), and BR analyses were run with random intercept only since the models with random slope rendered a warning for singular fit. All linear mixed-effects models were fitted with *lme4* package in R and confidence intervals and p-values were calculated with Wald statistics using the Satterthwaite approximation for denominator degrees of freedom. All linear mixed-effects models are specified in Supplementary Table 2. For each biomarker we performed a complete case analysis. We controlled for multiple comparisons using false discovery rate (FDR) correction within each outcome and clinical group (CU/MCI/AD dementia). Statistical significance was set at α < 0.05.

For understanding of additional explanatory power of each CSF biomarker and its interaction with tau, the marginal R^2^ (i.e. variance explained by the fixed effects only) and Akaike information criterion (AIC) were calculated for all models with and without the biomarker and with and without its interaction with tau (i.e. considering a model including tau load and all the covariates but not the biomarker or their interaction as benchmark).

Given the results in the CR analyses in the amyloid positive CU sample, where we found significant results for a wide range of CSF biomarkers, we performed an influential point analysis to investigate whether the results were driven by certain individuals. Analyses were re-run excluding one participant at a time, to identify individuals whose exclusion would produce a large change in the estimated coefficients. We thereafter also conducted the original CR analyses in the amyloid positive CU sample excluding the identified influential points.

### Multivariable models

Next, we tested which CSF proteins contributed most to CR or BR while controlling for all other CSF proteins. To that end, we performed Least Absolute Shrinkage and Selection Operator (LASSO) regressions including all biomarkers and their interactions with tau to evaluate a multivariable model in which all CSF markers are included simultaneously. LASSO regression models penalize the inclusion of weakly informative predictors for model selection [57], setting the coefficient of those predictors to 0. We used these models to investigate which biomarkers were retained in the model. We fitted the LASSO models as linear regressions (implemented in the *glmnet* package in R) with the annual change in atrophy (BR) or cognition (CR) as outcome variable. The annual change for each subject was estimated as the random slope from a linear mixed-effects model modelling atrophy or cognition over time in the total sample. The LASSO model included tau load and each individual biomarker alone and in interaction with tau as predictors. Age, sex, and mean NPX were included as covariates. The final samples for these models were determined by the availability of all CSF variables. Coefficients for covariates and tau were fixed so they could not be set to 0. We fitted these models separately for each diagnostic group. The regularization parameter lambda was determined by tenfold cross-validation for each model as the lambda that minimized the cost function. To assess the robustness of these results, we performed a bootstrapping procedure (2000 iterations), assessing at which proportion of the iterations each biomarker was selected in the model (i.e. given a weight different than 0).

## Results

Descriptive characteristics of the BR and CR samples are shown in Table 1. Due to data availability, the BR sample (*n* = 279) was smaller than the CR sample (*n* = 428). The number of participants also varied by CSF biomarker with some missing data for each variable (Supplementary Table 1). Mean follow-up time in the BR sample was 26.4 months (median 2 visits) and in the CR sample 25.1 months (median 3 visits; Supplementary Fig. 2). Mean age was 71.8 years in the BR sample and 72.1 years in the CR sample. The proportion of females was 51% and mean education level 12.6 years in both samples. In the BR sample, the association between temporal meta-ROI tau PET signal and AD signature cortical atrophy rate was significant in the whole sample (r = -0.59; *p* < 0.001; Supplementary Fig. 3) as well as within each diagnostic group (r -0.51 to -0.39; *p* < 0.001; shown graphically in Supplementary Fig. 3). In the CR sample, the associations in the whole sample between temporal meta-ROI tau PET signal and MMSE (r = -0.56; *p* < 0.001; Supplementary Fig. 4) and mPACC5 (r = -0.5; *p* < 0.001; Supplementary Fig. 4) were significant, as well as within each diagnostic group (r -0.55 to -0.38; *p* < 0.001; shown graphically in Supplementary Fig. 4).

**Table 1:** Descriptive characteristics

	Brain resilience sample (*n* = 279)	Cognitive resilience sample (*n* = 428)
Diagnosis n (%)		
Cognitively unimpaired	107 (38.4%)	134 (31.3%)
Mild cognitive impairment	82 (29.4%)	128 (29.9%)
Alzheimer’s disease dementia	90 (32.3%)	166 (38.8%)
Age, years	71.8 (7.5)	72.1 (7.6)
Sex, n (%) female	142 (51%)	218 (51%)
Education level, years	12.6 (4.2)	12.6 (4.3)
APOEε4 status, n (%) carriers	199 (72%)	300 (70%)
Temporal meta-ROI tau baseline, SUVR	1.63 (0.60)	1.70 (0.62)
Whole brain tau baseline, SUVR	1.31 (0.37)	1.35 (0.39)
AD-signature cortical thickness baseline, mm	2.56 (0.20)	2.54 (0.19)
AD-signature cortical atrophy rate, mm/year	-0.035 (0.038)	-
Whole brain cortical thickness baseline, mm	2.25 (0.11)	2.25 (0.11)
Whole brain cortical atrophy rate, mm/year	-0.017 (0.024)	-
MMSE baseline, points	25.6 (4.6)	25.1 (4.6)
MMSE annual change, points/year	-	-1.5 (2.3)
Follow-up, months	26.4 (10.7)	25.2 (11.4)
Follow-up, visits (median; range)	2; 2–4	3; 2–5
CSF mean NPX	0.614 (0.44)	0.630 (0.44)
CSF GFAP NPX	3.64 (1.18)	3.68 (1.16)
CSF GRN NPX	-1.44 (0.48)	-1.42 (0.48)
CSF ICAM-1 NPX	-3.38 (0.57)	-3.35 (0.57)
CSF IL-15 NPX	0.0115 (0.57)	0.0450 (0.60)
CSF TREM2 NPX	2.28 (0.93)	2.31 (0.92)
CSF YKL-40 NPX	5.67 (0.48)	5.68 (0.49)
CSF VEGF-A NPX	-1.06 (0.64)	-1.06 (0.65)
CSF VEGF-B NPX	0.0310 (0.58)	0.0581 (0.58)
CSF VEGF-C NPX	-0.380 (0.81)	-0.389 (0.81)
CSF VEGF-D NPX	-5.61 (0.73)	-5.62 (0.72)
CSF PGF NPX	0.569 (0.63)	0.609 (0.66)
CSF NRGN NPX	3.85 (0.91)	3.91 (0.93)
CSF NPTX2 NPX	4.82 (0.90)	4.79 (0.87)
CSF SV2A NPX	2.89 (0.69)	2.88 (0.750)
CSF SYT1 NPX	6.16 (0.65)	6.16 (0.65)
CSF 14–3-3 ζ/δ, fmol/μl	0.0896 (0.035)	0.0948 (0.040)
CSF NfL NPX	5.22 (0.97)	5.30 (0.97)
CSF NGF NPX	0.207 (0.12)	0.209 (0.118)

Descriptive characteristics of the brain resilience (BR) and cognitive resilience (CR) samples. Mean (SD) if not otherwise specified. Education level is missing for 5 participants in the BR sample and 15 participants in the CR sample, APOEε4 status is missing for 2 participants in both samples, and MMSE baseline and annual change are missing for 5 participants in the CR sample. *Abbreviations: ROI* region of interest, *SUVR* standardized uptake value ratio, *AD* Alzheimer’s disease, *MMSE* Mini Mental State Examination, *CSF* cerebrospinal fluid, *NPX* normalized protein expression, *GFAP* glial fibrillary acidic protein, *GRN* progranulin, *ICAM-1* intercellular adhesive molecule 1, *IL-15* interleukin 15, *TREM2* triggering receptor expressed on myeloid cells 2, *VEGF* vascular endothelial growth factor, *PGF* placental growth factor, *NRGN* neurogranin, *NPTX2* neuronal pentraxin 2, *SV2A* synaptic vesicle glycoprotein 2A, *SYT1* synaptotagmin 1, *NfL* neurofilament light; NGF – nerve growth factor

## Brain resilience in AD dementia

**Fig. 1:**
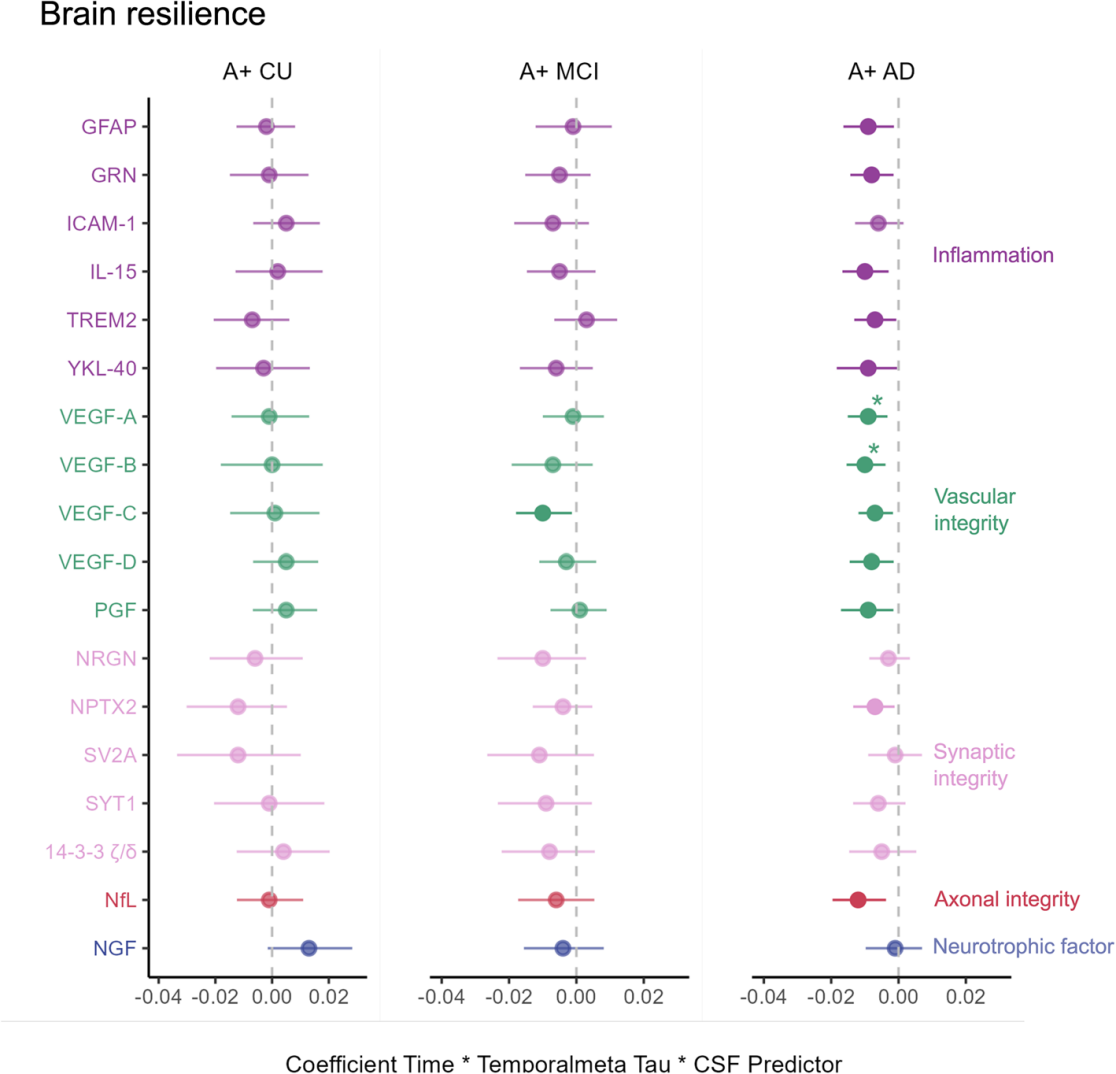
Moderating effects of CSF biomarkers on the association between tau levels and atrophy rate Linear mixed-effects models with longitudinal cortical thickness in AD signature cortex as outcome. Image shows the standardized coefficients for the interaction between each CSF biomarker and temporal meta-ROI tau with 95% CIs. A significant interaction indicates differing associations between tau PET signal and atrophy rate depending on the level of the CSF biomarker. VEGF-A and VEGF-B negatively moderate the association between tau PET signal and atrophy rate, indicating that at higher levels of these CSF biomarkers, the negative association between tau levels and atrophy rate is exacerbated. * p_FDR_ < 0.05; ** p_FDR_ < 0.01; *** p_FDR_ < 0.001. Abbreviations: CU – cognitively unimpaired; MCI – mild cognitive impairment; AD – Alzheimer’s disease; CI – confidence interval; GFAP – glial fibrillary acidic protein; GRN – progranulin; ICAM-1 – intercellular adhesive molecule 1; IL-15 – interleukin 15; TREM2 – triggering receptor expressed on myeloid cells 2; VEGF – vascular endothelial growth factor; PGF – placental growth factor; NRGN – neurogranin; NPTX2 – neuronal pentraxin 2; SV2A – synaptic vesicle glycoprotein 2A; SYT1 – synaptotagmin 1; NfL – neurofilament light; NGF – nerve growth factor; CSF – cerebrospinal fluid; FDR – false discovery rate

**Fig. 2:**
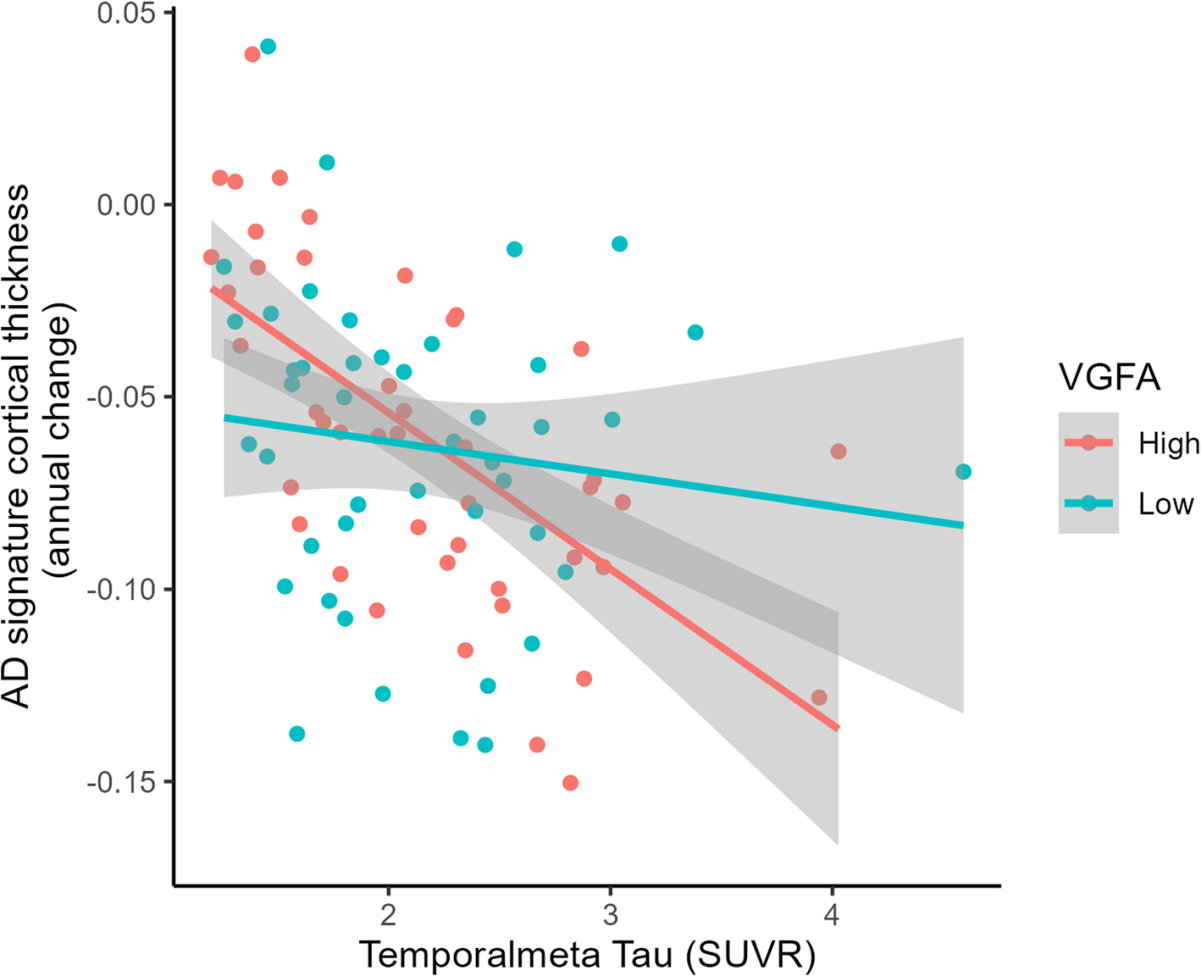
The association between tau levels and atrophy rate in AD dementia differs depending on levels of VEGF-A in CSF In the AD dementia group, the association between temporal meta-ROI tau signal (x axis) and atrophy rate of the AD signature cortex (y axis) is stronger in participants with higher baseline CSF levels of VEGF-A, here visualized by dividing the AD dementia sample by the median value of VEGF-A, and showing the associations for the low vs high group separately. Abbreviations: AD – Alzheimer’s disease; VEGF – vascular endothelial growth factor; CSF – cerebrospinal fluid; SUVR – standardized uptake value ratio

**Fig. 3:**
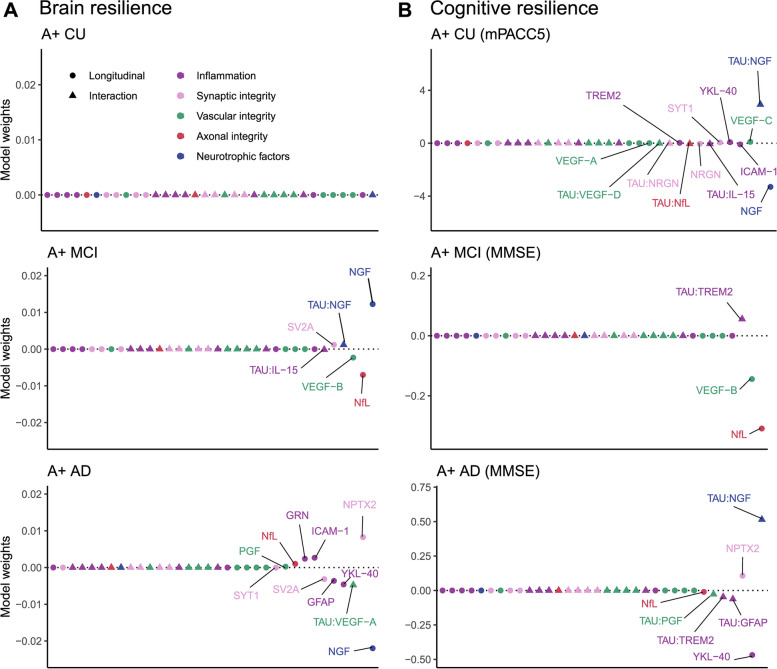
Multivariable LASSO regression models for investigating independent effects of different CSF biomarkers to brain and cognitive resilience LASSO regression models with AD signature atrophy rate as outcome for brain resilience analyses (panel A) and mPACC5 (A + CU) or MMSE (A + MCI and A + AD) slope as outcome for cognitive resilience analyses (panel B). The annotated variables were selected into the model. Model weights are shown along the y axis. Abbreviations: LASSO – Least Absolute Shrinkage and Selection Operator; CSF – cerebrospinal fluid; CU – cognitively unimpaired; mPACC5 – modified Preclinical Alzheimer's Cognitive Composite 5; MCI – mild cognitive impairment; MMSE – Mini Mental State Examination; AD – Alzheimer’s disease; CI – confidence interval; GFAP – glial fibrillary acidic protein; GRN – progranulin; ICAM-1 – intercellular adhesive molecule 1; IL-15 – interleukin 15; TREM2 – triggering receptor expressed on myeloid cells 2; VEGF – vascular endothelial growth factor; PGF – placental growth factor; NRGN – neurogranin; NPTX2 – neuronal pentraxin 2; SV2A – synaptic vesicle glycoprotein 2A; SYT1 – synaptotagmin 1; NfL – neurofilament light; NGF – nerve growth factor

In the AD dementia group, 12 CSF biomarkers significantly interacted with temporal meta-ROI tau PET signal in its association with longitudinal atrophy of the AD signature cortex such that the negative effect of tau was attenuated with lower levels of these proteins, with two markers of vascular integrity (VEGF-A (β = -0.009, p_FDR_ = 0.047) and VEGF-B (β = -0.010, p_FDR_ = 0.037)) surviving FDR correction (Table 2; Fig. 1). We exemplify these interactions graphically using VEGF-A in Fig. 2. For visualization purposes only, we divided the AD dementia sample into two groups using the median value of CSF VEGF-A, and we show the association between temporal meta-ROI tau PET signal and longitudinal atrophy for the low and high concentration groups separately (Fig. 2). The model including VEGF-A in interaction with tau PET also showed the highest increase in explained variance (ΔR2 = 8.8%; Supplementary Table 3) and largest decrease in AIC (ΔAIC = 13.3; Supplementary Table 3) compared to a model without the CSF biomarker. This was reflected in the LASSO regression predicting atrophy rate, where the interaction for VEGF-A with tau PET remained in the model with a negative estimate (Fig. 3a; Supplementary Table 4) and the finding was robust in it being selected into the model (i.e. having a coefficient other than 0) more than 60% of the bootstrap iterations (Supplementary Fig. 5). We found positive conditional main longitudinal effects (i.e. from the model with the interaction) for VEGF-B and NPTX2 (Supplementary Table 5) indicating a positive effect of these markers on brain structure over time, but because of our standardizing procedure with an amyloid negative CU reference group the interpretation of this is difficult since this reflects the effect of the CSF variable at tau levels equal to the ones in a CU individual without amyloid pathology and these analyses included only participants with AD dementia. In a model testing independent main effects (without the interaction term with tau), higher CSF VEGF-A concentration was associated with lower AD signature cortical thickness at baseline controlling for tau PET, also after FDR correction (β = -0.937, p_FDR_ = 0.037; Supplementary Table 6).

**Table 2:** Brain resilience

	A + CU			A + MCI			A + AD		
Variable	Std β coefficient (CI)	t	p	Std β coefficient (CI)	t	p	Std β coefficient (CI)	t	p
GFAP	-0.002 (-0.013 – 0.008)	-0.421	0.674	-0.001 (-0.012 – 0.011)	-0.140	0.889	-0.009 (-0.016 – -0.001)	-2.345	**0.021**
GRN	-0.001 (-0.015 – 0.013)	-0.145	0.885	-0.005 (-0.015 – 0.004)	-1.122	0.264	-0.008 (-0.014 – -0.001)	-2.446	**0.016**
ICAM-1	0.005 (-0.007 – 0.017)	0.859	0.392	-0.007 (-0.018 – 0.004)	-1.316	0.191	-0.006 (-0.013 – 0.001)	-1.575	0.118
IL-15	0.002 (-0.013 – 0.018)	0.318	0.751	-0.005 (-0.015 – 0.006)	-0.875	0.384	-0.010 (-0.017 – -0.003)	-2.833	**0.006**
TREM2	-0.007 (-0.021 – 0.006)	-1.074	0.285	0.003 (-0.007 – 0.012)	0.588	0.558	-0.007 (-0.013 – -0.001)	-2.194	**0.030**
YKL-40	-0.003 (-0.02 – 0.013)	-0.384	0.702	-0.006 (-0.017 – 0.005)	-1.093	0.277	-0.009 (-0.018 – 0)	-2.080	**0.040**
VEGF-A	-0.001 (-0.014 – 0.013)	-0.088	0.930	-0.001 (-0.01 – 0.008)	-0.196	0.845	-0.009 (-0.015 – -0.003)	-3.082	0.003*
VEGF-B	0 (-0.018 – 0.018)	-0.012	0.990	-0.007 (-0.019 – 0.005)	-1.186	0.238	-0.010 (-0.015 – -0.004)	-3.311	0.001*
VEGF-C	0.001 (-0.015 – 0.017)	0.122	0.903	-0.010 (-0.018 – -0.001)	-2.298	**0.023**	-0.007 (-0.012 – -0.002)	-2.624	**0.010**
VEGF-D	0.005 (-0.007 – 0.016)	0.824	0.411	-0.003 (-0.011 – 0.006)	-0.604	0.547	-0.008 (-0.015 – -0.001)	-2.424	**0.017**
PGF	0.005 (-0.007 – 0.016)	0.794	0.428	0.001 (-0.008 – 0.009)	0.152	0.879	-0.009 (-0.017 – -0.002)	-2.372	**0.019**
NRGN	-0.006 (-0.022 – 0.011)	-0.674	0.501	-0.010 (-0.023 – 0.003)	-1.551	0.124	-0.003 (-0.009 – 0.003)	-0.873	0.385
NPTX2	-0.012 (-0.03 – 0.005)	-1.390	0.167	-0.004 (-0.013 – 0.005)	-0.922	0.358	-0.007 (-0.013 – -0.001)	-2.360	**0.020**
SV2A	-0.012 (-0.034 – 0.01)	-1.060	0.291	-0.011 (-0.026 – 0.005)	-1.330	0.186	-0.001 (-0.009 – 0.007)	-0.255	0.799
SYT1	-0.001 (-0.02 – 0.018)	-0.103	0.918	-0.009 (-0.023 – 0.005)	-1.328	0.187	-0.006 (-0.013 – 0.002)	-1.455	0.149
14–3-3 ζ/δ	0.004 (-0.012 – 0.02)	0.473	0.637	-0.008 (-0.022 – 0.005)	-1.199	0.233	-0.005 (-0.015 – 0.005)	-0.936	0.352
NfL	-0.001 (-0.012 – 0.011)	-0.120	0.905	-0.006 (-0.017 – 0.005)	-1.049	0.296	-0.012 (-0.02 – -0.004)	-2.912	**0.004**
NGF	0.013 (-0.002 – 0.028)	1.773	0.078	-0.004 (-0.016 – 0.008)	-0.624	0.534	-0.001 (-0.01 – 0.007)	-0.326	0.745

Interaction effects with temporal meta-ROI tau (Time × Tau × Variable β) on longitudinal AD signature cortical atrophy across all diagnostic groups. A significant interaction indicates differing associations between tau PET signal and atrophy rate depending on the level of the CSF biomarker, with a negative interaction meaning that the negative association between tau PET signal and atrophy rate is exacerbated at higher levels of the CSF biomarker. * p_FDR_ < 0.05; ** p_FDR_ < 0.01; *** p_FDR_ < 0.001. *Abbreviations: AD* Alzheimer’s disease, *CU* cognitively unimpaired, *MCI* mild cognitive impairment, *CI* confidence interval, *GFAP* glial fibrillary acidic protein, *GRN* progranulin, *ICAM-1* intercellular adhesive molecule 1, *IL-15* interleukin 15, *TREM2* triggering receptor expressed on myeloid cells 2, *VEGF* vascular endothelial growth factor, *PGF* placental growth factor, *NRGN* neurogranin, *NPTX2* neuronal pentraxin 2, *SV2A* synaptic vesicle glycoprotein 2A, *SYT1* synaptotagmin 1, *NfL* neurofilament light, *NGF* nerve growth factor, *ROI* region of interest, *PET* positron emission tomography, *FDR* false discovery rate

### Brain resilience in MCI

In the MCI group, no moderation effect was significant after FDR correction (Table 2; Fig. 1). However, levels of NfL were associated with longitudinal cortical atrophy (β = -0.109, p_FDR_ = 0.033) as well as baseline cortical thickness (β = -0.708, p_FDR_ = 0.033) when controlling for tau PET signal, also after FDR correction (Supplementary tables 6–7). The model including NfL also had a higher R2 (ΔR2 = 14.8%) and lower AIC (ΔAIC = 21.3) than the one without (Supplementary Table 3). In the LASSO regression, NfL was also retained in the model with a negative estimate, indicating a negative association with atrophy rate controlling for tau PET signal (Fig. 3a; Supplementary Table 4).

### Brain resilience in CU

In the CU group, no significant interaction between any of the proteins and tau PET signal was observed, and no results from the models without interaction terms survived FDR correction (Table 2; Supplementary tables 6–7; Fig. 1).

### Secondary analyses of brain resilience

In our secondary analyses with whole brain tau PET uptake as predictor in the model and whole brain cortical thickness as outcome, no significant interaction effects or main longitudinal or cross-sectional associations were seen after FDR correction (Supplementary tables 8–10).

### Cognitive resilience in CU

**Fig. 4:**
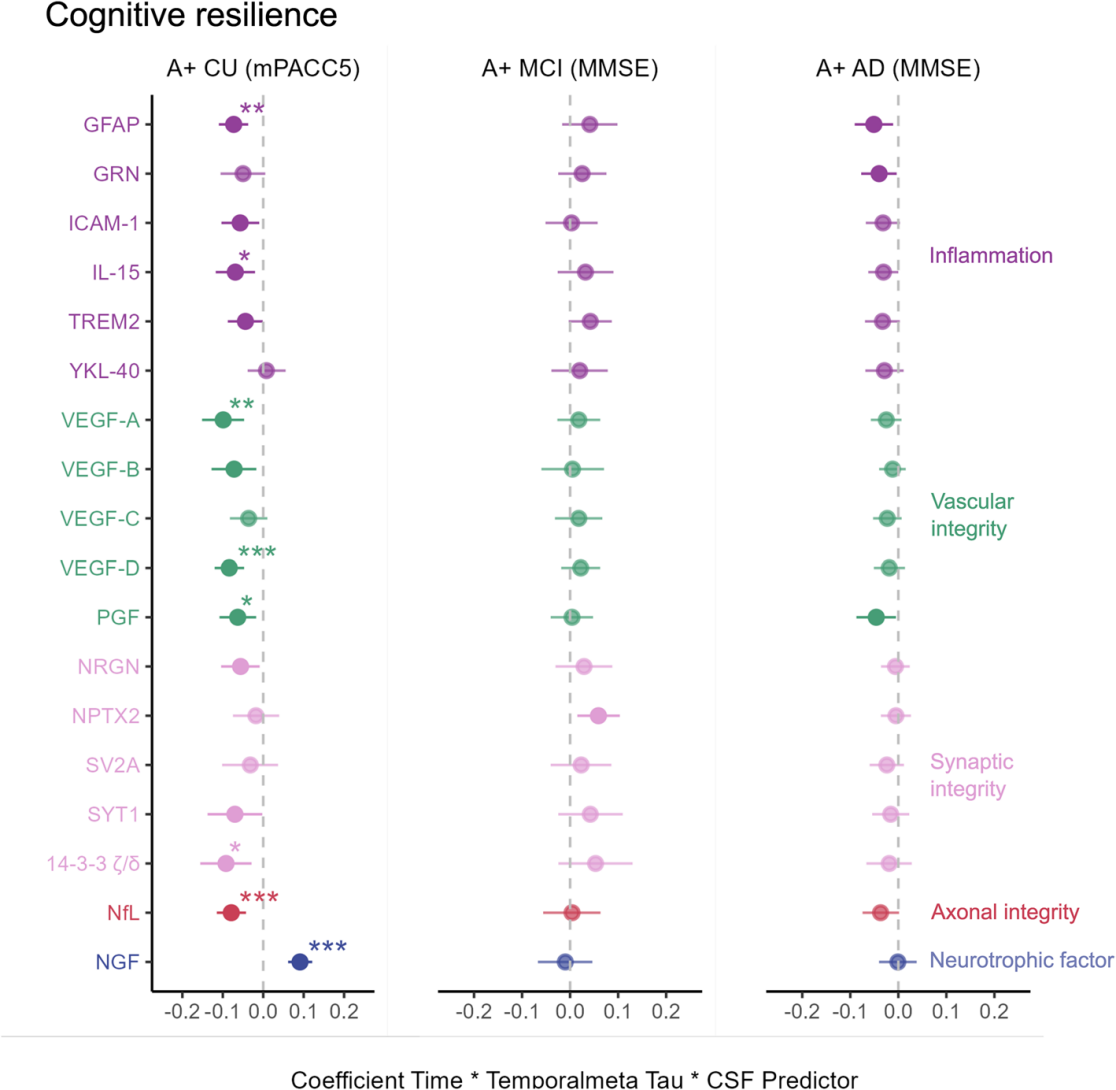
Moderating effects of CSF biomarkers on the association between tau levels and cognitive decline Linear mixed-effects models with longitudinal mPACC5 (A + CU) or MMSE (A + MCI and A + AD) as outcome. Image shows the standardized coefficients for the interaction between each CSF biomarker and temporal meta-ROI tau with 95% CIs. A significant interaction indicates differing associations between tau PET signal and cognitive decline depending on the level of the CSF biomarker, with a negative interaction meaning that the negative association between tau PET signal and cognitive decline is exacerbated at higher levels of the CSF biomarker. * p_FDR_ < 0.05; ** p_FDR_ < 0.01; *** p_FDR_ < 0.001. Abbreviations: CU – cognitively unimpaired; mPACC5 – modified Preclinical Alzheimer's Cognitive Composite 5; MCI – mild cognitive impairment; MMSE – Mini Mental State Examination; AD – Alzheimer’s disease; CI – confidence interval; GFAP – glial fibrillary acidic protein; GRN – progranulin; ICAM-1 – intercellular adhesive molecule 1; IL-15 – interleukin 15; TREM2 – triggering receptor expressed on myeloid cells 2; VEGF – vascular endothelial growth factor; PGF – placental growth factor; NRGN – neurogranin; NPTX2 – neuronal pentraxin 2; SV2A – synaptic vesicle glycoprotein 2A; SYT1 – synaptotagmin 1; NfL – neurofilament light; NGF – nerve growth factor; CSF – cerebrospinal fluid; FDR – false discovery rate

Significant interactions with temporal meta-ROI tau PET signal were observed in the CU group for 13 CSF proteins with 8 surviving FDR correction (Table 3; Fig. 4). Inflammatory (GFAP (β = -0.073, p_FDR_ = 0.001) and IL-15 (β = -0.069, p_FDR_ = 0.045)), vascular (VEGF-A (β = -0.099, p_FDR_ = 0.003), VEGF-D (β = -0.084, p_FDR_ < 0.001), and PGF (β = -0.063, p_FDR_ = 0.046)), and synaptic biomarkers (14–3-3 ζ/δ (β = -0.092, p_FDR_ = 0.041), as well as levels of NfL (β = -0.079, p_FDR_ < 0.001) moderated the association between temporal meta-ROI tau PET signal and cognitive decline over time such that lower levels of these proteins attenuated the negative effect of tau on cognitive decline over time. Also NGF (β = 0.079, p_FDR_ < 0.001) interacted with tau PET signal, but instead higher levels of NGF attenuated the negative effect of tau on cognitive decline. The interaction effects for NGF (ΔAIC = 38.8; ΔR2 = 13.6%), NfL (ΔAIC = 12.8; ΔR2 = 2.9%), and VEGF-D (ΔAIC = 13.4; ΔR2 = 2.9%) improved the model fit and explanatory power the most compared to its benchmark models (Supplementary Table 11). Given the large number of significant results across different groups of biomarkers, we performed an influential data point analysis to investigate whether the results were driven by certain individuals. This rendered three participants deemed as influential, and when excluding those from the analyses, the results were no longer statistically significant in the CU group. Since these participants’ results in specific variables are plausible they were retained in the main analyses but warrant careful interpretation of the results.

**Table 3:** Cognitive resilience

	A + CU (mPACC5)			A + MCI (MMSE)			A + AD (MMSE)			
Variable	Std β coefficient (CI)	t	p	Std β coefficient (CI)	t	p		Std β coefficient (CI)	t	p
GFAP	-0.073 (-0.11 – -0.037)	-4.001	< 0.001**	0.041 (-0.017 – 0.099)	1.411	0.161		-0.051 (-0.091 – -0.011)	-2.512	**0.013**
GRN	-0.05 (-0.105 – 0.005)	-1.794	0.075	0.025 (-0.025 – 0.076)	1.004	0.317		-0.04 (-0.077 – -0.004)	-2.163	**0.032**
ICAM-1	-0.057 (-0.103 – -0.01)	-2.383	**0.018**	0.003 (-0.051 – 0.057)	0.111	0.912		-0.032 (-0.068 – 0.004)	-1.780	0.077
IL-15	-0.069 (-0.118 – -0.02)	-2.797	0.006*	0.032 (-0.026 – 0.09)	1.096	0.275		-0.031 (-0.063 – 0)	-1.976	0.050
TREM2	-0.044 (-0.088 – -0.001)	-2.024	**0.045**	0.042 (-0.003 – 0.087)	1.843	0.068		-0.033 (-0.069 – 0.003)	-1.810	0.073
YKL-40	0.008 (-0.038 – 0.055)	0.357	0.722	0.02 (-0.039 – 0.078)	0.666	0.507		-0.029 (-0.069 – 0.011)	-1.433	0.155
VEGF-A	-0.099 (-0.151 – -0.047)	-3.774	< 0.001**	0.018 (-0.026 – 0.063)	0.802	0.424		-0.025 (-0.057 – 0.007)	-1.570	0.119
VEGF-B	-0.072 (-0.128 – -0.017)	-2.581	**0.011**	0.005 (-0.06 – 0.071)	0.165	0.869		-0.012 (-0.04 – 0.015)	-0.879	0.381
VEGF-C	-0.036 (-0.082 – 0.01)	-1.535	0.127	0.018 (-0.032 – 0.067)	0.715	0.476		-0.023 (-0.053 – 0.007)	-1.520	0.131
VEGF-D	-0.084 (-0.12 – -0.047)	-4.527	< 0.001***	0.022 (-0.018 – 0.063)	1.078	0.283		-0.019 (-0.051 – 0.014)	-1.135	0.259
PGF	-0.063 (-0.108 – -0.017)	-2.728	0.007*	0.004 (-0.04 – 0.048)	0.164	0.870		-0.046 (-0.087 – -0.005)	-2.218	**0.028**
NRGN	-0.056 (-0.104 – -0.009)	-2.336	**0.021**	0.029 (-0.03 – 0.088)	0.964	0.337		-0.006 (-0.036 – 0.024)	-0.409	0.683
NPTX2	-0.018 (-0.075 – 0.039)	-0.613	0.541	0.059 (0.015 – 0.103)	2.656	**0.009**		-0.005 (-0.036 – 0.026)	-0.320	0.750
SV2A	-0.032 (-0.102 – 0.037)	-0.931	0.354	0.023 (-0.04 – 0.086)	0.713	0.477		-0.024 (-0.06 – 0.012)	-1.334	0.184
SYT1	-0.07 (-0.138 – -0.002)	-2.044	**0.043**	0.042 (-0.025 – 0.11)	1.254	0.212		-0.016 (-0.054 – 0.023)	-0.810	0.420
14–3-3 ζ/δ	-0.092 (-0.156 – -0.029)	-2.873	0.005*	0.053 (-0.024 – 0.13)	1.357	0.177		-0.019 (-0.066 – 0.028)	-0.807	0.421
NfL	-0.079 (-0.115 – -0.043)	-4.292	< 0.001***	0.004 (-0.056 – 0.063)	0.119	0.905		-0.037 (-0.075 – 0.002)	-1.901	0.060
NGF	0.091 (0.062 – 0.121)	6.052	< 0.001***	-0.01 (-0.067 – 0.046)	-0.361	0.719		-0.001 (-0.04 – 0.038)	-0.052	0.959

Interaction effects with temporal meta-ROI tau (Time × Tau × Variable β) on longitudinal global cognition across all diagnostic groups. A significant interaction indicates differing associations between tau PET signal and cognitive decline depending on the level of the CSF biomarker, with a negative interaction meaning that the negative association between tau PET signal and cognitive decline is exacerbated at higher levels of the CSF biomarker. * p_FDR_ < 0.05; ** p_FDR_ < 0.01; *** p_FDR_ < 0.001. *Abbreviations:* CU cognitively unimpaired, *mPACC5* – modified Preclinical Alzheimer's Cognitive Composite 5, *MCI* mild cognitive impairment, MMSE – Mini Mental State Examination, *AD* Alzheimer’s disease, *CI* confidence interval, GFAP – glial fibrillary acidic protein, *GRN* progranulin, *ICAM-1* intercellular adhesive molecule 1, *IL-15* interleukin 15, *TREM2* triggering receptor expressed on myeloid cells 2, *VEGF* vascular endothelial growth factor, *PGF* placental growth factor, *NRGN* neurogranin, *NPTX2* neuronal pentraxin 2, *SV2A* synaptic vesicle glycoprotein 2A, *SYT1* synaptotagmin 1, *NfL* neurofilament light, *NGF* nerve growth factor, *ROI* region of interest, *PET* positron emission tomography, *FDR* false discovery rate

In the LASSO regression, we observed a similar pattern as in the bivariate analyses with the interaction term with tau PET remaining in the model for one inflammatory (IL-15; negative estimate), one vascular (VEGF-D; negative estimate), and one synaptic (neurogranin; negative estimate) biomarker as well as the interactions for NfL (negative estimate) and NGF (positive estimate; Fig. 3b; Supplementary Table 12). The bootstrap results showed that the interactions with tau for these five biomarkers were most often selected into the model, except that VEGF-C was the more selected vascular biomarker rather than VEGF-D (Supplementary Fig. 6).

No significant main longitudinal or cross-sectional associations were found in the CU group when controlling for tau PET (Supplementary tables 13–15).

### Cognitive resilience in MCI

In the MCI group, no moderation effect was significant after FDR correction (Table 3; Fig. 4). However, levels of NfL was associated with faster cognitive decline over time controlling for tau PET signal also after FDR correction ((β = -0.690, p_FDR_ = 0.025), Supplementary Table 13).

### Cognitive resilience in AD dementia

In the AD dementia group, no moderation effect was significant after FDR correction (Table 3; Fig. 4).

### Secondary analyses of cognitive resilience

In our secondary analyses looking at interaction effects with, and main effects controlling for, whole brain tau PET signal, similar results were found for the CU group as in the primary analyses (Supplementary tables 16–18). However, unlike in the primary analyses we also saw significant interaction effects in the AD dementia group, with inflammatory (GRN and ICAM-1; β -0.088 to -0.079, p_FDR_ 0.028–0.047) and vascular (PGF; β = -0.094, p_FDR_ = 0.013) biomarkers moderating the association between whole brain tau PET signal and global cognition assessed with MMSE(Supplementary Table 16).

With ADAS-Cog immediate recall as outcome, we found no significant interactions with temporal meta-ROI tau PET signal and no longitudinal or cross-sectional main effects for any CSF biomarkers surviving FDR correction (Supplementary tables 19–21). For TMTA, however, a similar pattern as for mPACC5 and MMSE was found in the CU group (Supplementary Table 22) with significant interaction effects for inflammatory (GFAP), vascular (VEGF-A, VEGF-B, VEGF-D, and PGF), and synaptic biomarkers (NRGN) as well as NfL and NGF. No significant main longitudinal or cross-sectional associations were found when controlling for tau PET after FDR correction (Supplementary tables 23–24).

Since the CR sample (because of data availability) included participants that were not included in the BR sample, a sub-analysis within the CR sample including only participants also included in the BR sample was also performed. From the 279 participants in the BR sample, 275 participants (106 CU, 81 MCI, 88 AD dementia) had available longitudinal cognitive data and were therefore eligible for the CR analyses. The results were consistent with the initial analyses, with many significant interaction terms in the amyloid positive CU group, but not in the MCI and AD dementia groups (Supplementary Table 25).

## Discussion

In this longitudinal study of people across the AD spectrum, we tested how inflammatory, vascular, synaptic, axonal, and neurotrophic CSF biomarkers influence the association of tau PET levels with atrophy and cognitive decline. We found strongest evidence for effects of biomarkers of vascular and axonal integrity, where higher concentrations of these biomarkers were associated with faster than expected cortical atrophy or cognitive decline, given the level of tau PET signal. This suggests that these biological processes influence disease progression in AD by either providing or counteracting resilience against tau pathology.

One of the most robust findings of our study was the interactive effect of VEGF-A and VEGF-B with tau on AD signature cortical atrophy in the AD dementia group. The association between tau PET uptake and atrophy rate was attenuated at lower levels of these CSF proteins. Lower levels of VEGF-A were also associated with higher cortical thickness relative to the amount of tau pathology at baseline. Our results also indicate that at lower levels of VEGF-A and VEGF-D the detrimental effect of tau pathology on cognitive decline is attenuated in amyloid positive CU individuals, although these results were not as robust and need to be interpreted with caution as described in the results section. Proteins from the VEGF family are involved in the angiogenesis and homeostasis of brain vasculature [58] as well as neural development [59], and expression increases in response to hypoxia [60]. Previous studies have shown a negative association between VEGF-B expression and cognitive trajectory [41], which is in line with our findings. There is also an association between VEGF variants and radiological findings of cerebrovascular pathology such as white matter lesions [42], which in turn are associated with both cognitive decline [61] and lower brain resilience [24]. However, there are some inconsistencies in the literature where for example one study looking at CU, MCI, and AD dementia participants from the Alzheimer's Disease Neuroimaging Initiative (ADNI) showed that higher VEGF levels in CSF was associated with better cognitive trajectory and slower hippocampal atrophy [62]. A possible explanation for differing findings between cohorts could be that ADNI has a strong focus on AD, with only limited presence of vascular co-pathology. The role of VEGF proteins could potentially be different depending on the context, i.e. whether it is increased in response to substantial vascular pathology or not. Another recent study investigating the role of plasma levels of VEGFs in a CU sample found significant interaction effects with amyloid levels with differing results within the VEGF family; lower levels of VEGF-A and higher levels of PGF were associated with greater cognitive decline in participants with higher amyloid levels [63]. This is in line with our results for PGF, but the opposite of what we found for VEGF-A. The differing results could be due to plasma levels of VEGF-A not reflecting only the integrity of the brain vasculature but also vascular alterations in other organs since it is produces by many different cell types.

Lower levels of NfL were consistently associated with slower atrophy rate and better cognitive performance relative to the amount of cerebral tau pathology. In MCI participants, we found a negative association between levels of NfL and longitudinal atrophy, baseline cortical thickness, and longitudinal global cognition, controlling for tau. In CU participants, lower levels of NfL attenuated the negative effect of tau on global cognition, and in AD dementia participants, lower NfL levels attenuated the negative effect of tau on AD signature cortical atrophy, although this finding did not survive FDR correction. This is at large in line with previous literature, where higher NfL levels are associated with greater atrophy and worse cognitive decline [50, 64], even though its interactive effect with tau is not as well established. NfL is viewed as a non-specific marker of axonal neurodegeneration and can be increased due to many different underlying disease processes [65]. As we have shown in another BioFINDER cohort [29], NfL confers information complementary to structural MRI (another marker of neurodegeneration) in predicting cognitive changes.

We found interactive effects with tau pathology for CSF proteins associated with neuroinflammatory processes in the amyloid positive CU group when assessing CR. Similar effects were implicated in the AD dementia group when assessing BR, although not significant after FDR correction. Other studies have shown that higher GFAP levels in CSF are associated with worse cognitive performance [36] and that the levels are higher in CU individuals with more amyloid and tau pathology pathology [35]. Two neuropathology studies comparing AD cases with “resilient” cases (persons with substantial amounts of AD pathology but no dementia diagnosis), show lower levels of GFAP in the resilient cases compared to AD cases [66, 67]. In the central nervous system, IL-15 is expressed by glial cells as well as neurons [68] and it is involved in activation of T cells and natural killer cells [69]. The levels of IL-15 in CSF are increased in amyloid positive subjects across the AD continuum compared with amyloid negative subjects, and higher levels are associated with higher CSF phosphorylated tau and clinical progression [40, 70]. Other cytokines have been studied in the context of resilience using neuropathology showing differential expression of cytokines in the entorhinal cortex in AD dementia patients compared to “resilient” cases (i.e. persons without dementia but with considerable amounts of AD pathology in the brain) [71]. Regarding TREM2, genetic studies have shown that variants in the TREM2 gene are associated with higher risk for AD [72], and higher levels of CSF AD biomarkers [73]. Biomarker studies are inconclusive, where increased levels of TREM2 in CSF have been observed in AD patients compared to controls [37] but in another study an association between higher levels and attenuated cognitive decline in AD individuals was observed [38]. In the context of resilience, one study using neuropathology showed higher TREM2 expression and better preserved axonal/dendritic structure in “resilient” individuals compared to AD subjects [74].

Among the CSF proteins reflective of synaptic integrity, the only finding surviving correction for multiple comparisons was the negative interaction between temporal meta-ROI tau PET and 14–3-3 ζ/δ on cognitive decline in the CU group. Proteins from the 14–3-3 family are increased in CSF in AD subjects compared to controls [54, 75, 76] and are also established biomarkers of Creutzfeldt-Jakob disease, a disease with rapidly progressing neurodegeneration [77]. Higher levels also increase the risk of conversion from MCI to dementia [76], which could be interpreted as people with lower 14–3-3 ζ/δ levels being more resilient. For NPTX2, a protein previously shown to be found at lower concentrations in AD subjects compared to controls [54] and associated with better outcome in the context of AD [45], we found diverging results, with higher levels enhancing the negative effect of tau on atrophy in the AD group, but both in models with and without the interaction term with tau, higher levels was instead associated with less atrophy over time and higher cortical thickness at baseline controlling for tau, and in MCI subjects, higher NPTX2 levels attenuated the negative effects of tau on longitudinal cognition.

Higher levels of NGF were associated with better cognitive trajectories, with attenuation of the negative effect of tau pathology on global cognition in the CU group, and slower cognitive decline controlling for tau PET levels in the AD dementia group (and also for CU and MCI participants in our secondary analyses using whole brain tau levels as tau measure). Also in the BR analyses higher NGF levels were associated with lower atrophy rate controlling for tau in the AD dementia group. Together these results suggest a protective role for NGF. NGF is important for development and maintenance of the peripheral nervous system, but also for cholinergic neurons in the central nervous system, and clinical trials have even tested its therapeutic effects in AD [78].

Our main findings of interactive effects with tau PET were different across the AD clinical spectrum with significant interactions in the AD dementia group for brain resilience and in the CU group for cognitive resilience, although to some extent similar biomarkers were implicated. This could indicate that processes such as loss of vascular and axonal integrity as well as inflammation contribute to early functional alterations exacerbating cognitive decline without evidence of atrophy, but at later disease stages it is also associated with faster atrophy rate. The lack of interaction effects with temporal meta-ROI tau on cognition in the AD group could be due to the strong effect of regional tau pathology on cognitive decline in this group and therefore levels of other biomarkers do not add enough information to significantly moderate the association between tau and cognition. The differing results between clinical groups could also be due to methodological issues, such as low variance in atrophy rate relative to premorbid differences in brain structure in the CU group, making it harder to capture a moderating effect on atrophy in this group.

## Strengths and limitations

Strengths of this study include the longitudinal design and the representation of, and division into, different cognitive stages of the AD continuum. The use of PET as biomarker of tau pathology instead of CSF is also a strength, considering inter-individual differences in CSF dynamics and the recent findings of the importance of the overall protein concentrations when using fluid biomarkers [79–81]. This study also has several limitations. First, the follow-up time of around two years is relatively short, especially in CU participants. Second, the relatively small group of MCI subjects, especially in the BR sample, increases the risk of false negative findings in this group. A larger sample size overall could also enable us to categorize participants along the resilience spectrum and to specifically compare properties of the participants in the extremes at each end of the spectrum (i.e. highly resilient and highly vulnerable participants). Third, the predictive effects of the CSF biomarkers are hard to interpret since the association between higher levels and faster progression could also be due to the participants with higher levels being further along the AD trajectory rather than the biological process or pathology in itself contributing to progression. To some extent this was controlled for by including tau PET levels in the models and thus controlling for disease stage, but the intricate interplay between AD biomarkers and the included biomarkers of interest still makes the interpretation from a resilience perspective difficult. Fourth, it is important to again emphasize that the results in CU participants regarding cognitive resilience were influenced by participants with high tau and fast cognitive decline. We decided not to exclude these participants since they represent a group with high baseline tau but still normal cognition and therefore are prime examples of highly resilient individuals (i.e., the main focus of this study) and their values in different variables are plausible. Lastly, our results are limited to one cohort which is ethnically homogeneous, and findings need to be replicated in other settings to ensure generalizability.

## Conclusions

Biomarkers of co-existing pathological processes, in particular vascular pathology and axonal degeneration, interact with levels of tau pathology on its effects on the downstream effects of AD pathology, indicating that these processes could play a role in the phenomena referred to as brain and cognitive resilience.

## Supplementary Information

Supplementary Material 1.

## Data Availability

Pseudonymized data will be shared by request from a qualified academic investigator for the sole purpose of replicating procedures and results presented in the article and as long as data transfer is in agreement with EU legislation on the general data protection regulation and decisions by the Swedish Ethical Review Authority and Region Skåne, which should be regulated in a material transfer agreement.

## Abbreviations

Aβ: Amyloid-β
AD: Alzheimer’s disease
ADAS-Cog: Alzheimer Disease Assessment Scale-Cognitive Subscale
AIC: Akaike information criterion
BR: Brain resilience
CR: Cognitive resilience
CSF: Cerebrospinal fluid
CU: Cognitively unimpaired
DNA: Deoxyribonucleic acid
DSM: Diagnostic and Statistical Manual of Mental Disorders
FDG: Fluorodeoxyglucose
FDR: False discovery rate
GFAP: Glial fibrillary acidic protein
GRN: Progranulin
ICAM-1: Intercellular adhesion molecule 1
IL-15: Interleukin 15
LASSO: Least Absolute Shrinkage and Selection Operator
MCI: Mild cognitive impairment
MRI: Magnetic resonance imaging
PACC: Preclinical Alzheimer Cognitive Composite
MMSE: Mini Mental State Examination
NfL: Neurofilament light
NGF: Nerve growth factor
NPTX2: Neuronal pentraxin 2
NPX: Normalized protein expression
NRGN: Neurogranin
PCR: Polymerase chain reaction
PET: Positron emission tomography
PGF: Placental growth factor
ROI: Region of interest
SUVR: Standardized uptake value ratio
SV2A: Synaptic vesicle glycoprotein 2A
SYT1: Synaptotagmin 1
TMTA: Trailmaking test A
TREM2: Triggering receptor expressed on myeloid cells 2
VEGF-A: Vascular endothelial growth factor A
VEGF-B: Vascular endothelial growth factor B
VEGF-C: Vascular endothelial growth factor C
VEGF-D: Vascular endothelial growth factor D
YKL-40: Chitinase-3-like protein 1
14–3-3 ζ/δ: 14-3–3 Zeta/delta
