# Supplementary material for: Biological mechanisms of resilience to tau pathology in Alzheimer’s disease
Source: Alzheimers Res Ther. 2024 Oct 12;16:221. doi: 10.1186/s13195-024-01591-9 (PMC11470552; doi:10.1186/s13195-024-01591-9)
Supplement: Supplementary file 1 — Supplementary Material 1. [file 13195_2024_1591_MOESM1_ESM.pdf]

Supplementary information

Supplementary figures

Supplementary figure 1. Histograms of CSF biomarkers in the cognitive resilience sample. Olink variables in NPX units, 14-3-3 z/d log2 transformed.

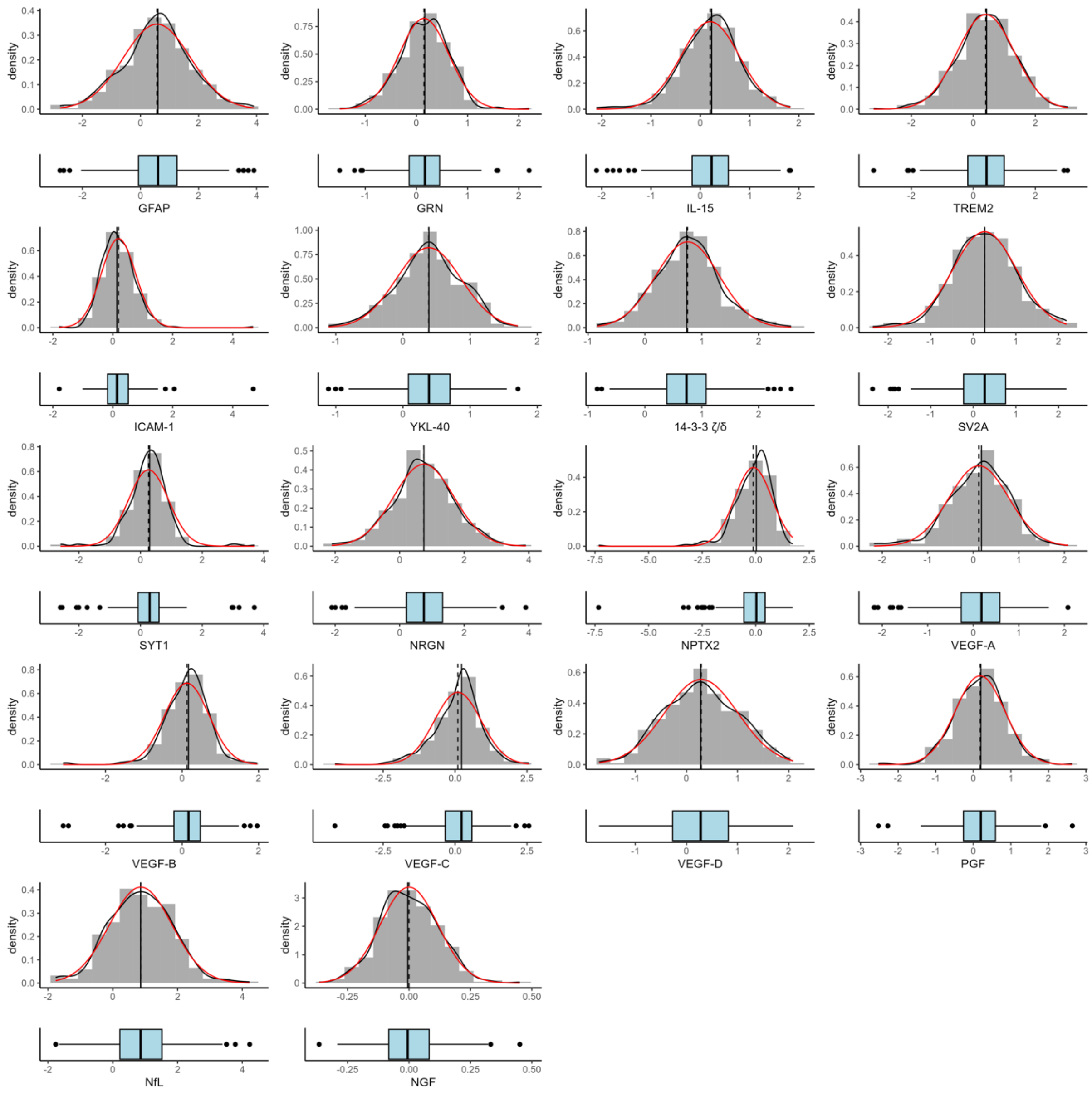

Supplementary figure 2. Timepoints for cognitive assessment visits for each participant in the cognitive resilience sample.

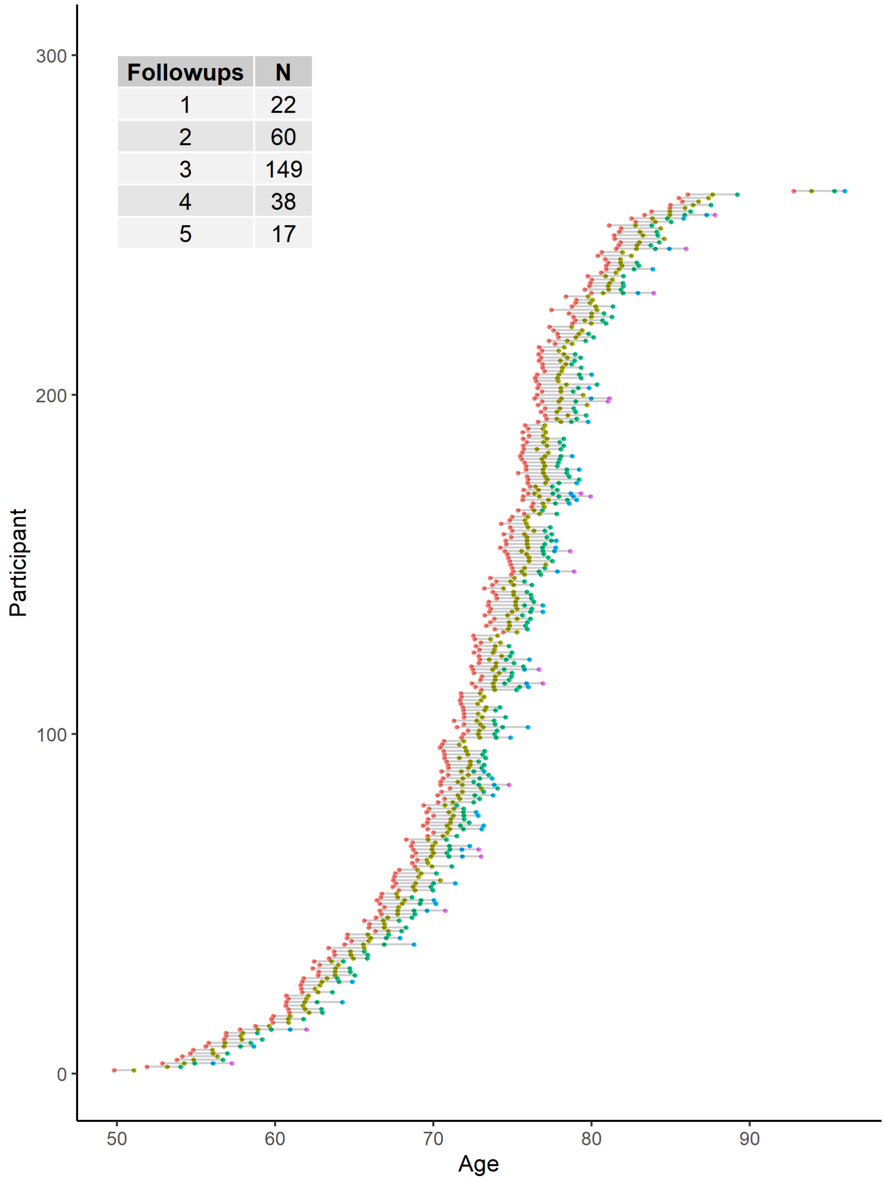

Supplementary figure 3. The relationship between tau levels and atrophy rate.

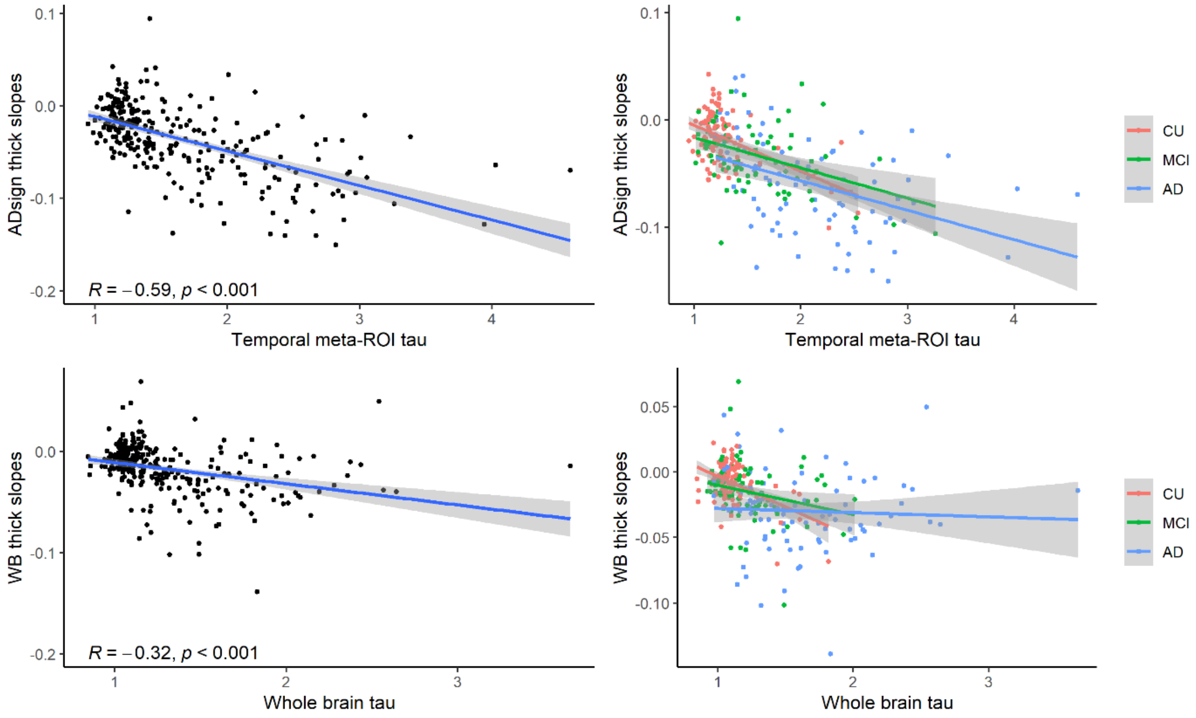

The relationship between tau levels and atrophy rate in the AD signature cortex (top panels) and whole brain cortex (bottom panels) in the whole brain resilience sample (left panels) and divided by cognitive status (right panels).

Supplementary figure 4. The relationship between temporal meta-ROI tau levels and cognitive decline.

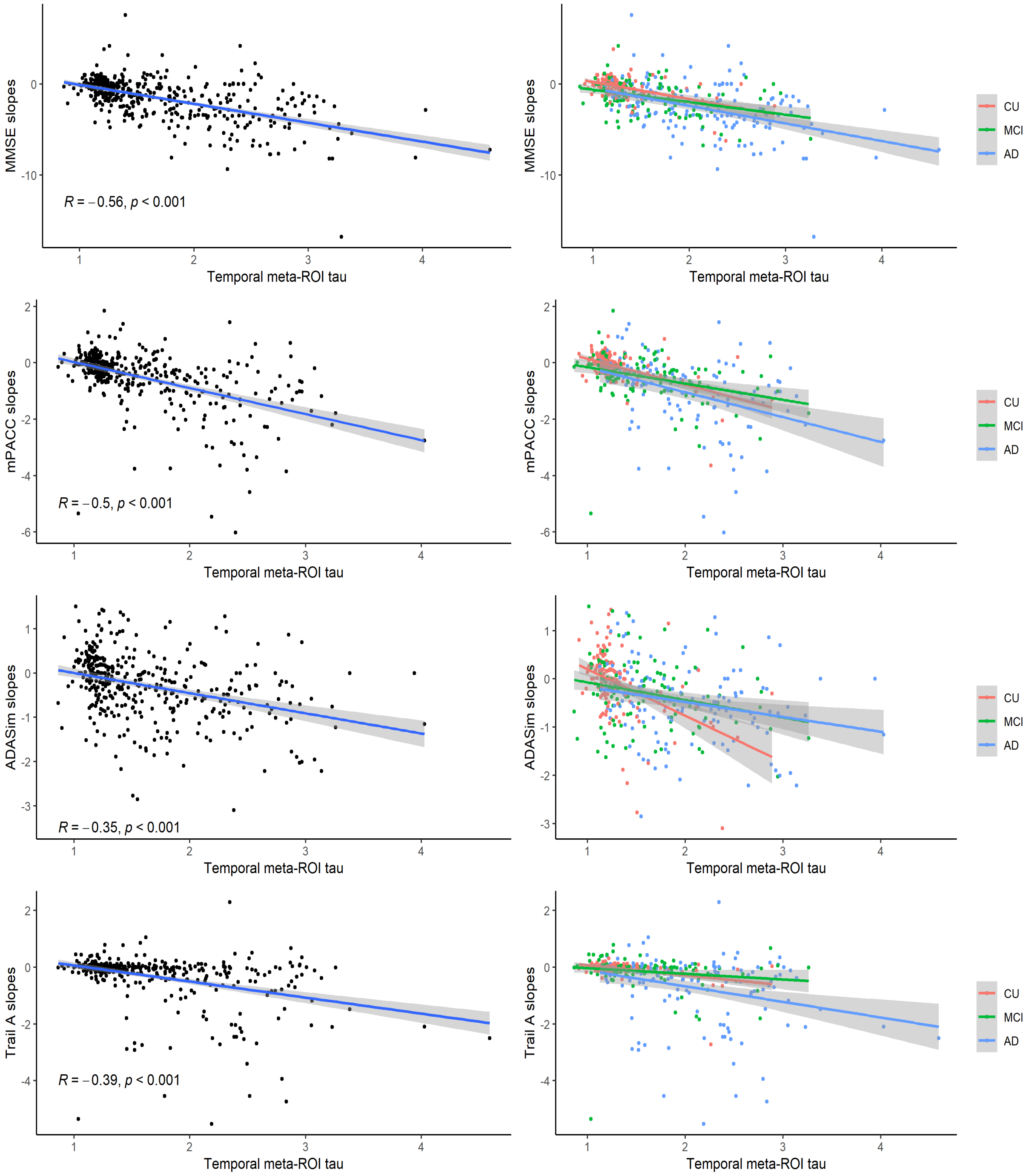

The relationship between temporal meta-ROI tau levels and cognitive decline in different cognitive tests in the whole cognitive resilience sample (left panels) and divided by cognitive status (right panels).

Supplementary figure 5. LASSO bootstrapping procedure in the brain resilience sample with AD signature cortical atrophy rate as outcome.

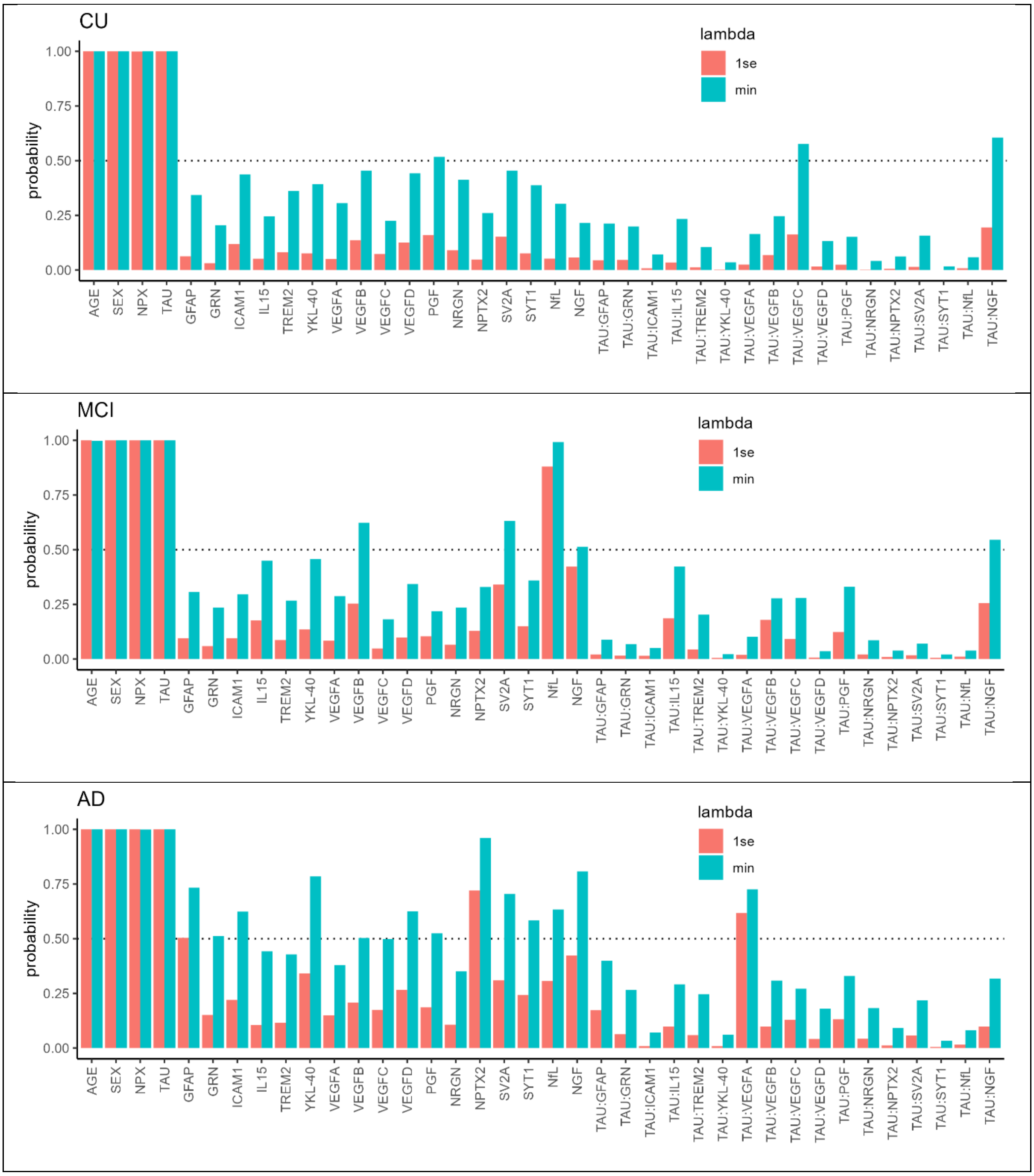

Results from the LASSO bootstrapping procedure in the brain resilience sample with AD signature cortical atrophy rate as outcome. The y axis shows at which proportion of the 2000 iterations a variable was selected into the model (i.e. given a weight different than 0). Lambda refers to a penalty term introduced to the regression model, used to set coefficients of variables that do not add to the model to 0, to avoid overfitting. Results from analyses with different values of lambda are presented, where min means at minimum lambda and 1se means minimum lambda plus 1 SE.

Supplementary figure 6. LASSO bootstrapping procedure in the cognitive resilience sample with mPACC5 slope as outcome.

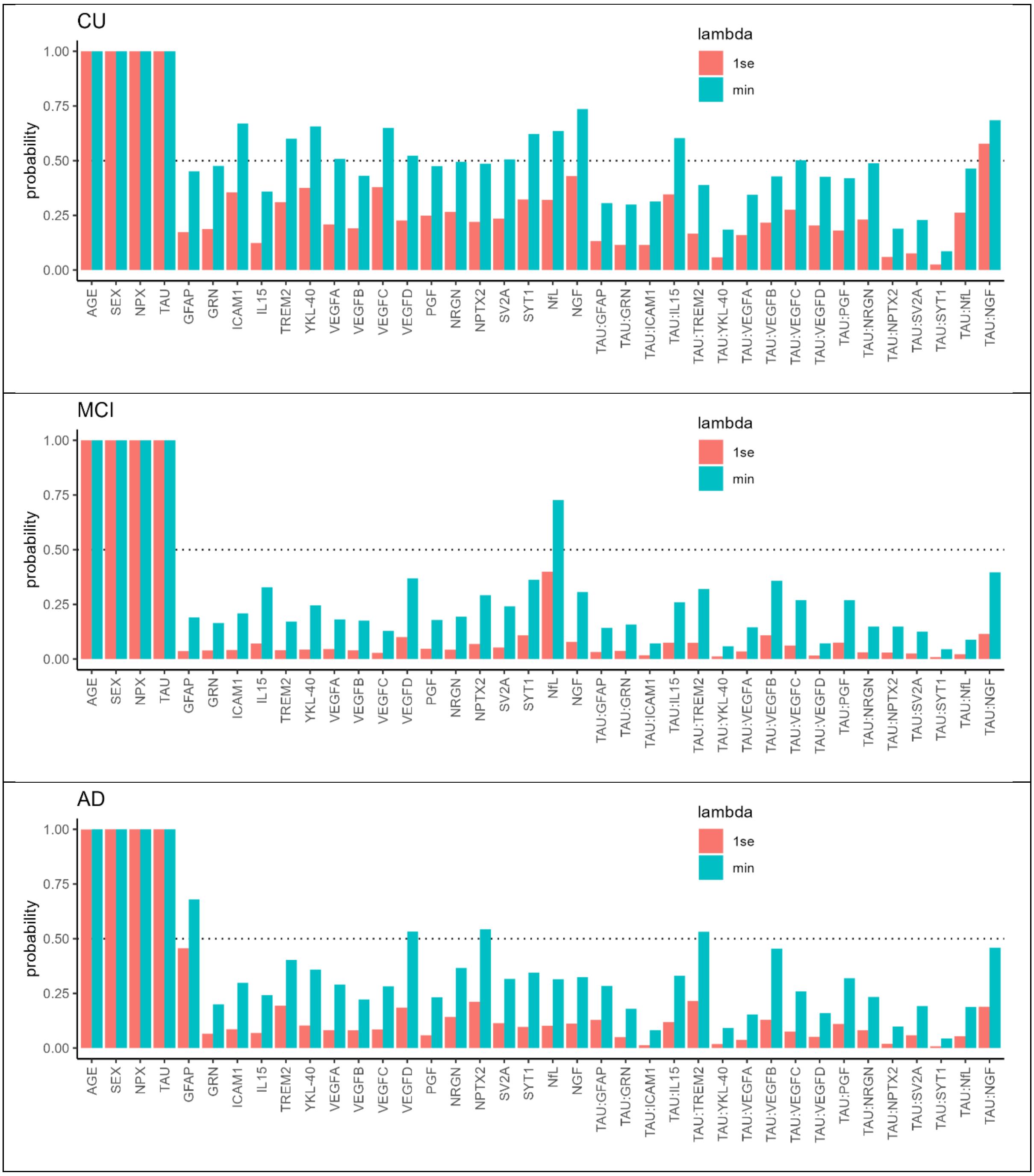

Results from the LASSO bootstrapping procedure in the cognitive resilience sample with mPACC5 slope as outcome. The y axis shows at which proportion of the 2000 iterations a variable was selected into the model (i.e. given a weight different than 0). Lambda refers to a penalty term introduced to the regression model, used to set coefficients of variables that do not add to the model to 0, to avoid overfitting. Results from analyses with different values of lambda are presented, where min means at minimum lambda and 1se means minimum lambda plus 1 SE.

Supplementary tables

Supplementary table 1. Missing CSF data, percentage of missing values across the CSF markers in the cognitive resilience sample.

	A+ CU (n=134)	A+ MCI (n=128)	A+ AD (n=166)
Mean NPX	5.37	4.67	4.91
GFAP	5.84	5.37	5.84
GRN	5.61	4.91	5.14
ICAM-1	5.37	4.67	5.14
IL-15	6.07	4.91	5.37
TREM2	6.54	5.14	4.91
YKL-40	5.37	4.67	5.14
VEGF-A	5.84	5.14	4.91
VEGF-B	6.78	7.48	6.78
VEGF-C	5.84	5.61	5.14
VEGF-D	5.84	5.14	4.91
PGF	5.84	5.14	4.91
NRGN	5.37	5.14	5.14
NPTX2	5.61	4.91	4.91
SV2A	5.84	5.37	6.07
SYT1	5.84	4.91	5.14
14-3-3 ζ/δ	7.01	8.88	8.41
NfL	5.61	4.91	5.84
NGF	5.37	4.91	5.14

Supplementary table 2. Model descriptions of all linear mixed-effects models.

	Statistical model	Reported coefficient(s)
Table 2	AD signature cortical thickness ~<br>~ Time + Tau_{temp} + CSF marker + Age + Sex + Mean CSF +<br>+ Time* Tau_{temp} + Time*CSF marker + Time*Age + Time*Sex + Time*Mean CSF + Tau_{temp}*CSF marker +<br>+ Time* Tau_{temp}*CSF marker +<br>+ (1 | ID)	Time* Tau_{temp}*CSF marker
Table 3	Cognitive performance (mPACC5/MMSE) ~<br>~ Time + Tau_{temp} + CSF marker + Age + Sex + Mean CSF +<br>+ Time* Tau_{temp} + Time*CSF marker + Time*Age + Time*Sex + Time*Mean CSF + Tau_{temp}*CSF marker +<br>+ Time* Tau_{temp}*CSF marker +<br>+ (Time | ID)	Time* Tau_{temp}*CSF marker
Supplementary table 5	AD signature cortical thickness ~<br>~ Time + Tau_{temp} + CSF marker + Age + Sex + Mean CSF +<br>+ Time*Tau_{temp} + Time*CSF marker + Time*Age + Time*Sex + Time*Mean CSF + Tau_{temp}*CSF marker +<br>+ Time*Tau_{temp}*CSF marker +<br>+ (1 | ID)	CSF marker and Time*CSF marker
Supplementary table 6	AD signature cortical thickness ~<br>~ Time + Tau_{temp} + CSF marker + Age + Sex + Mean CSF +<br>+ Time*Tau_{temp} + Time*CSF marker + Time*Age + Time*Sex + Time*Mean CSF + Tau_{temp}*CSF marker +<br>+ (1 | ID)	Time*CSF marker
Supplementary table 7	AD signature cortical thickness ~<br>~ Time + Tau_{temp} + CSF marker + Age + Sex + Mean CSF +<br>+ Time*Tau_{temp} + Time*CSF marker + Time*Age + Time*Sex + Time*Mean CSF + Tau_{temp}*CSF marker +<br>+ (1 | ID)	CSF marker
Supplementary table 8	Whole brain cortical thickness ~<br>~ Time + $Tau_{wholebrain}$ + CSF marker + Age + Sex + Mean CSF +<br>+ Time*$Tau_{wholebrain}$ + Time*CSF marker + Time*Age + Time*Sex + Time*Mean CSF + $Tau_{wholebrain}$*CSF marker +<br>+ Time*$Tau_{wholebrain}$*CSF marker +<br>+ (1 | ID)	Time*$Tau_{wholebrain}$*CSF marker

Supplementary table 9	Whole brain cortical thickness ~<br>~ Time + $Tau_{wholebrain}$ + CSF marker + Age + Sex + Mean CSF +<br>+ Time*$Tau_{wholebrain}$ + Time*CSF marker + Time*Age + Time*Sex + Time*Mean CSF + $Tau_{wholebrain}$*CSF marker +<br>+ (1 | ID)	Time*CSF marker
Supplementary table 10	Whole brain cortical thickness ~<br>~ Time + $Tau_{wholebrain}$ + CSF marker + Age + Sex + Mean CSF +<br>+ Time*$Tau_{wholebrain}$ + Time*CSF marker + Time*Age + Time*Sex + Time*Mean CSF + $Tau_{wholebrain}$*CSF marker +<br>+ (1 | ID)	CSF marker
Supplementary table 13	Cognitive performance (mPACC5/MMSE) ~<br>~ Time + Tau_{temp} + CSF marker + Age + Sex + Mean CSF +<br>+ Time*Tau_{temp} + Time*CSF marker + Time*Age + Time*Sex + Time*Mean CSF + Tau_{temp}*CSF marker +<br>+ (Time | ID)	Time*CSF marker
Supplementary table 14	Cognitive performance (mPACC5/MMSE) ~<br>~ Time + Tau_{temp} + CSF marker + Age + Sex + Mean CSF +<br>+ Time*Tau_{temp} + Time*CSF marker + Time*Age + Time*Sex + Time*Mean CSF + Tau_{temp}*CSF marker +<br>+ (Time | ID)	CSF marker
Supplementary table 15	Cognitive performance (mPACC5) ~<br>~ Time + Tau_{temp} + CSF marker + Age + Sex + Mean CSF +<br>+ Time*Tau_{temp} + Time*CSF marker + Time*Age + Time*Sex + Time*Mean CSF + Tau_{temp}*CSF marker +<br>+ Time*Tau_{temp}*CSF marker +<br>+ (Time | ID)	CSF marker and Time*CSF marker
Supplementary table 16	Cognitive performance (mPACC5/MMSE) ~<br>~ Time + $Tau_{wholebrain}$ + CSF marker + Age + Sex + Mean CSF +<br>+ Time*$Tau_{wholebrain}$ + Time*CSF marker + Time*Age + Time*Sex + Time*Mean CSF + $Tau_{wholebrain}$*CSF marker +<br>+ Time*$Tau_{wholebrain}$*CSF marker +<br>+ (Time | ID)	Time*$Tau_{wholebrain}$*CSF marker
Supplementary table 17	Cognitive performance (mPACC5/MMSE) ~<br>~ Time + $Tau_{wholebrain}$ + CSF marker + Age + Sex + Mean CSF +<br>+ Time*$Tau_{wholebrain}$ + Time*CSF marker + Time*Age + Time*Sex + Time*Mean CSF + $Tau_{wholebrain}$*CSF marker +<br>+ (Time | ID)	Time*CSF marker
Supplementary table 18	Cognitive performance (mPACC5/MMSE) ~<br>~ Time + $Tau_{wholebrain}$ + CSF marker + Age + Sex + Mean CSF +	CSF marker

	+ Time*$Tau_{wholebrain}$ + Time*CSF marker + Time*Age + Time*Sex + Time*Mean CSF + $Tau_{wholebrain}$*CSF marker +<br>+ (Time | ID)	
Supplementary table 19	Cognitive performance (ADAS-Cog immediate recall) ~<br>~ Time + Tau_{temp} + CSF marker + Age + Sex + Mean CSF +<br>+ Time*Tau_{temp} + Time*CSF marker + Time*Age + Time*Sex + Time*Mean CSF + Tau_{temp}*CSF marker +<br>+ Time*Tau_{temp}*CSF marker +<br>+ (Time | ID)	Time*Tau_{temp}*CSF marker
Supplementary table 20	Cognitive performance (ADAS-Cog immediate recall) ~<br>~ Time + Tau_{temp} + CSF marker + Age + Sex + Mean CSF +<br>+ Time*Tau_{temp} + Time*CSF marker + Time*Age + Time*Sex + Time*Mean CSF + Tau_{temp}*CSF marker +<br>+ (Time | ID)	Time*CSF marker
Supplementary table 21	Cognitive performance (ADAS-Cog immediate recall) ~<br>~ Time + Tau_{temp} + CSF marker + Age + Sex + Mean CSF +<br>+ Time*Tau_{temp} + Time*CSF marker + Time*Age + Time*Sex + Time*Mean CSF + Tau_{temp}*CSF marker +<br>+ (Time | ID)	CSF marker
Supplementary table 22	Cognitive performance (TMTA) ~<br>~ Time + Tau_{temp} + CSF marker + Age + Sex + Mean CSF +<br>+ Time*Tau_{temp} + Time*CSF marker + Time*Age + Time*Sex + Time*Mean CSF + Tau_{temp}*CSF marker +<br>+ Time*Tau_{temp}*CSF marker +<br>+ (1 | ID)	Time*Tau_{temp}*CSF marker
Supplementary table 23	Cognitive performance (TMTA) ~<br>~ Time + Tau_{temp} + CSF marker + Age + Sex + Mean CSF +<br>+ Time*Tau_{temp} + Time*CSF marker + Time*Age + Time*Sex + Time*Mean CSF + Tau_{temp}*CSF marker +<br>+ (1 | ID)	Time*CSF marker
Supplementary table 24	Cognitive performance (TMTA) ~<br>~ Time + Tau_{temp} + CSF marker + Age + Sex + Mean CSF +<br>+ Time*Tau_{temp} + Time*CSF marker + Time*Age + Time*Sex + Time*Mean CSF + Tau_{temp}*CSF marker +<br>+ (1 | ID)	CSF marker
Supplementary table 25	Cognitive performance (mPACC5/MMSE) ~<br>~ Time + Tau_{temp} + CSF marker + Age + Sex + Mean CSF +<br>+ Time*Tau_{temp} + Time*CSF marker + Time*Age + Time*Sex + Time*Mean CSF + Tau_{temp}*CSF marker +<br>+ Time*Tau_{temp}*CSF marker +<br>+ (Time | ID)	Time*Tau_{temp}*CSF marker

Supplementary table 3. Model performance metrics of the brain resilience analyses.

		A+ CU						A+ MCI						A+ AD					
Variable		AIC	ΔAIC	R2	ΔR2	N (subj)	N (obs)	AIC	ΔAIC	R2	ΔR2	N (subj)	N (obs)	AIC	ΔAIC	R2	ΔR2	N (subj)	N (obs)
GFAP	Benchmark	-660.48	0.00	54.80	0.00	94	245	-398.39	0.00	17.88	0.00	76	193	-356.62	0.00	37.10	0.00	86	191
	Main	-659.19	-1.28	55.00	0.20	94	245	-396.76	-1.63	18.18	0.30	76	193	-357.19	0.58	38.07	0.97	86	191
	Interaction	-658.42	-2.06	55.14	0.33	94	245	-393.80	-4.59	18.93	1.05	76	193	-359.87	3.25	39.24	2.14	86	191
GRN	Benchmark	-649.07	0.00	51.36	0.00	94	244	-406.81	0.00	18.29	0.00	78	197	-365.26	0.00	37.28	0.00	88	195
	Main	-645.12	-3.95	51.37	0.00	94	244	-412.60	5.79	24.84	6.55	78	197	-361.34	-3.92	37.29	0.02	88	195
	Interaction	-641.61	-7.46	51.17	-0.19	94	244	-412.68	5.87	27.63	9.35	78	197	-365.04	-0.22	38.71	1.44	88	195
ICAM-1	Benchmark	-659.99	0.00	51.43	0.00	95	247	-406.81	0.00	18.29	0.00	78	197	-360.84	0.00	37.15	0.00	87	193
	Main	-658.44	-1.55	51.76	0.33	95	247	-406.49	-0.32	19.48	1.19	78	197	-357.22	-3.63	37.20	0.05	87	193
	Interaction	-655.75	-4.24	51.61	0.18	95	247	-405.33	-1.48	20.52	2.23	78	197	-357.69	-3.15	38.72	1.57	87	193
IL-15	Benchmark	-644.88	0.00	51.46	0.00	93	242	-407.43	0.00	17.03	0.00	77	193	-358.24	0.00	39.79	0.00	86	190
	Main	-641.30	-3.58	51.65	0.19	93	242	-408.14	0.71	21.76	4.73	77	193	-354.73	-3.52	39.85	0.07	86	190
	Interaction	-637.84	-7.04	51.54	0.08	93	242	-404.98	-2.45	21.75	4.72	77	193	-359.83	1.59	41.00	1.21	86	190
TREM2	Benchmark	-625.03	0.00	50.59	0.00	92	237	-407.43	0.00	17.03	0.00	77	193	-365.26	0.00	37.28	0.00	88	195
	Main	-624.93	-0.10	51.25	0.66	92	237	-405.78	-1.65	17.10	0.07	77	193	-363.72	-1.54	38.58	1.30	88	195
	Interaction	-623.81	-1.22	52.78	2.19	92	237	-403.05	-4.38	17.93	0.90	77	193	-367.19	1.93	40.60	3.33	88	195
YKL-40	Benchmark	-659.99	0.00	51.43	0.00	95	247	-406.81	0.00	18.29	0.00	78	197	-360.84	0.00	37.15	0.00	87	193
	Main	-659.22	-0.77	51.90	0.47	95	247	-402.89	-3.92	18.40	0.11	78	197	-363.16	2.32	39.40	2.25	87	193
	Interaction	-655.88	-4.11	52.41	0.99	95	247	-400.07	-6.73	18.46	0.17	78	197	-365.24	4.40	40.59	3.44	87	193
VEGF-A	Benchmark	-650.86	0.00	50.95	0.00	94	244	-407.43	0.00	17.03	0.00	77	193	-365.26	0.00	37.28	0.00	88	195
	Main	-647.49	-3.37	50.99	0.04	94	244	-407.55	0.12	20.30	3.27	77	193	-371.92	6.66	44.75	7.48	88	195
	Interaction	-644.75	-6.10	50.93	-0.02	94	244	-405.45	-1.98	22.24	5.21	77	193	-378.54	13.28	46.08	8.80	88	195
VEGF-B	Benchmark	-635.05	0.00	54.32	0.00	91	237	-378.84	0.00	19.24	0.00	69	178	-345.71	0.00	37.38	0.00	84	187
	Main	-633.08	-1.97	54.76	0.44	91	237	-379.19	0.35	21.06	1.82	69	178	-344.71	-1.00	38.13	0.75	84	187
	Interaction	-639.08	4.02	58.78	4.46	91	237	-377.00	-1.84	21.35	2.10	69	178	-351.42	5.71	38.65	1.28	84	187
VEGF-C	Benchmark	-649.66	0.00	52.55	0.00	93	241	-398.39	0.00	17.88	0.00	76	193	-365.26	0.00	37.28	0.00	88	195
	Main	-652.55	2.89	54.72	2.17	93	241	-396.72	-1.68	20.43	2.56	76	193	-361.43	-3.83	37.38	0.10	88	195
	Interaction	-656.29	6.64	59.14	6.59	93	241	-398.14	-0.25	20.70	2.82	76	193	-365.39	0.13	38.60	1.32	88	195
VEGF-D	Benchmark	-650.86	0.00	50.95	0.00	94	244	-407.43	0.00	17.03	0.00	77	193	-365.26	0.00	37.28	0.00	88	195
	Main	-648.16	-2.70	51.76	0.81	94	244	-404.82	-2.61	18.20	1.18	77	193	-361.86	-3.41	37.43	0.15	88	195
	Interaction	-652.10	1.24	53.59	2.64	94	244	-401.66	-5.77	18.59	1.56	77	193	-366.69	1.42	39.82	2.54	88	195
PGF	Benchmark	-650.86	0.00	50.95	0.00	94	244	-407.43	0.00	17.03	0.00	77	193	-365.26	0.00	37.28	0.00	88	195
	Main	-648.56	-2.29	51.03	0.08	94	244	-407.43	0.00	18.68	1.66	77	193	-362.18	-3.08	37.80	0.53	88	195
	Interaction	-651.39	0.53	52.59	1.64	94	244	-405.06	-2.37	19.70	2.67	77	193	-368.88	3.62	41.57	4.30	88	195
NRGN	Benchmark	-659.99	0.00	51.43	0.00	95	247	-396.25	0.00	17.82	0.00	77	193	-355.70	0.00	37.32	0.00	87	192
	Main	-659.01	-0.99	52.65	1.22	95	247	-394.91	-1.33	19.76	1.94	77	193	-355.68	-0.01	39.69	2.37	87	192
	Interaction	-656.05	-3.95	53.23	1.80	95	247	-393.30	-2.95	19.87	2.04	77	193	-352.44	-3.26	39.65	2.33	87	192
NPTX2	Benchmark	-645.00	0.00	51.08	0.00	94	243	-406.81	0.00	18.29	0.00	78	197	-365.26	0.00	37.28	0.00	88	195
	Main	-641.47	-3.53	51.12	0.03	94	243	-403.74	-3.06	18.55	0.26	78	197	-373.77	8.51	42.60	5.33	88	195
	Interaction	-641.87	-3.14	52.86	1.77	94	243	-400.60	-6.20	18.62	0.33	78	197	-375.66	10.40	42.98	5.71	88	195
SV2A	Benchmark	-660.09	0.00	51.88	0.00	93	242	-394.68	0.00	17.48	0.00	76	192	-360.21	0.00	36.30	0.00	87	193
	Main	-656.41	-3.68	51.89	0.01	93	242	-398.84	4.16	25.03	7.55	76	192	-356.51	-3.69	36.57	0.27	87	193
	Interaction	-653.87	-6.22	51.83	-0.05	93	242	-396.67	1.99	25.26	7.78	76	192	-353.23	-6.98	37.15	0.86	87	193

SYT1	Benchmark	-653.96	0.00	51.45	0.00	94	245	-401.60	0.00	18.00	0.00	77	195	-365.26	0.00	37.28	0.00	88	195
	Main	-650.93	-3.03	51.55	0.09	94	245	-399.13	-2.47	19.15	1.15	77	195	-361.98	-3.28	37.29	0.02	88	195
	Interaction	-648.04	-5.92	51.61	0.16	94	245	-397.78	-3.82	19.82	1.82	77	195	-361.03	-4.23	38.03	0.75	88	195
14-3-3 ζ/δ	Benchmark	-590.12	0.00	50.27	0.00	85	224	-355.46	0.00	19.46	0.00	66	171	-321.17	0.00	32.33	0.00	78	175
	Main	-586.67	-3.44	50.29	0.03	85	224	-353.91	-1.56	20.62	1.16	66	171	-319.05	-2.11	32.51	0.18	78	175
	Interaction	-588.49	-1.63	51.55	1.28	85	224	-352.88	-2.58	22.27	2.81	66	171	-316.13	-5.03	32.57	0.24	78	175
NFL	Benchmark	-659.99	0.00	51.43	0.00	95	247	-403.14	0.00	17.74	0.00	77	195	-350.41	0.00	37.13	0.00	86	190
	Main	-656.04	-3.95	51.47	0.04	95	247	-424.47	21.33	32.57	14.83	77	195	-347.17	-3.24	37.48	0.35	86	190
	Interaction	-655.43	-4.57	51.65	0.22	95	247	-422.45	19.31	33.50	15.76	77	195	-352.93	2.52	38.65	1.51	86	190
NGF	Benchmark	-659.99	0.00	51.43	0.00	95	247	-397.39	0.00	18.32	0.00	77	194	-365.26	0.00	37.28	0.00	88	195
	Main	-660.50	0.51	51.82	0.39	95	247	-397.69	0.30	21.02	2.70	77	194	-361.92	-3.34	37.46	0.19	88	195
	Interaction	-661.60	1.61	52.58	1.16	95	247	-394.78	-2.62	22.24	3.92	77	194	-358.36	-6.90	37.62	0.34	88	195

Supplementary table 4. Results from brain resilience LASSO regressions.

	A+ CU (n=81)	A+ MCI (n=61)	A+ AD (n=76)
Age	-0.00053	-0.00046	-0.00082
Sex	-0.0015	-0.0020	-0.011
Mean NPX	0.0028	0.0064	0.014
Tau	-0.040	-0.0095	-0.020
GFAP	0	0	-0.0036
GRN	0	0	0.0024
ICAM-1	0	0	0.0027
IL-15	0	0	0
TREM2	0	0	0
YKL-40	0	0	-0.0046
VEGF-A	0	0	0
VEGF-B	0	-0.0023	0
VEGF-C	0	0	0
VEGF-D	0	0	0
PGF	0	0	0.00023
NRGN	0	0	0
NPTX2	0	0	0.0083
SV2A	0	0.0012	-0.0032
SYT1	0	0	$-5.24*10^{-5}$
NfL	0	-0.0070	0.0010
NGF	0	0.012	-0.022
GFAP*tau	0	0	0
GRN*tau	0	0	0
ICAM-1*tau	0	0	0
IL-15*tau	0	-0.00013	0
TREM2*tau	0	0	0
YKL-40*tau	0	0	0
VEGF-A*tau	0	0	-0.0048
VEGF-B*tau	0	0	0
VEGF-C*tau	0	0	0
VEGF-D*tau	0	0	0
PGF*tau	0	0	0
NRGN*tau	0	0	0
NPTX2*tau	0	0	0

SV2A*tau	0	0	0
SYT1*tau	0	0	0
NfL*tau	0	0	0
NGF*tau	$2.5*10^{-8}$	0.0012	0

Estimates from the LASSO regression in the brain resilience sample with AD signature cortex atrophy rate as outcome.

Supplementary table 5. Conditional effects (dependent on the interaction term with tau) on AD signature cortical atrophy in the AD dementia group.

Brain resilience (BR); conditional main cross-sectional and longitudinal effects on AD signature cortical atrophy in the AD dementia group						
	Conditional main cross-sectional effect			Conditional main longitudinal effect		
Variable	Std β coefficient (CI)	t	p	Std β coefficient (CI)	t	p
GFAP	0.028 (-0.595 – 0.651)	0.089	0.929	0.029 (-0.1 – 0.157)	0.441	0.66
GRN	0.161 (-0.579 – 0.901)	0.433	0.666	0.051 (-0.098 – 0.2)	0.681	0.497
ICAM-1						
IL-15	0.021 (-0.475 – 0.517)	0.084	0.934	0.126 (0.02 – 0.231)	2.363	**0.02**
TREM2	-0.125 (-0.635 – 0.385)	-0.488	0.627	0.085 (-0.016 – 0.187)	1.664	0.099
YKL-40	-0.288 (-0.917 – 0.34)	-0.91	0.365	0.177 (0.044 – 0.31)	2.63	**0.01**
VEGF-A	-0.819 (-1.472 – -0.166)	-2.492	**0.014**	0.117 (-0.027 – 0.261)	1.615	0.109
VEGF-B	0.163 (-0.429 – 0.755)	0.548	0.585	0.215 (0.094 – 0.335)	3.535	**0.001***
VEGF-C	0.054 (-0.411 – 0.518)	0.229	0.819	0.08 (-0.018 – 0.178)	1.621	0.108
VEGF-D	0.181 (-0.342 – 0.705)	0.688	0.493	0.06 (-0.053 – 0.174)	1.055	0.294
PGF	0.175 (-0.351 – 0.701)	0.662	0.51	0.111 (0.001 – 0.222)	2.003	**0.048**
NRGN						
NPTX2	0.529 (0.029 – 1.029)	2.101	**0.038**	0.179 (0.07 – 0.288)	3.259	**0.001***
SV2A						
SYT1						
14-3-3 ζ/δ						
NfL	0.22 (-0.214 – 0.655)	1.006	0.317	0.043 (-0.046 – 0.131)	0.958	0.34
NGF						

* $p_{FDR} < 0.05$; ** $p_{FDR} < 0.01$; *** $p_{FDR} < 0.001$

Supplementary table 6. Main longitudinal effects of CSF proteins on AD signature atrophy controlling for temporal meta-ROI tau.

Brain resilience (BR); main longitudinal effects (Time × Variable β) on AD signature cortical atrophy across all diagnostic groups controlling for temporal meta-ROI tau									
	A+ CU			A+ MCI			A+ AD		
Variable	Std β coefficient (CI)	t	p	Std β coefficient (CI)	t	p	Std β coefficient (CI)	t	p
GFAP	-0.032 (-0.074 – 0.01)	-1.514	0.132	0.047 (-0.017 – 0.11)	1.463	0.146	-0.084 (-0.173 – 0.004)	-1.886	0.062
GRN	-0.008 (-0.082 – 0.066)	-0.218	0.828	0.058 (-0.012 – 0.129)	1.635	0.105	-0.02 (-0.161 – 0.121)	-0.285	0.776
ICAM-1	-0.039 (-0.09 – 0.012)	-1.508	0.134	0.037 (-0.009 – 0.082)	1.596	0.113	0.027 (-0.076 – 0.13)	0.516	0.607
IL-15	-0.004 (-0.05 – 0.043)	-0.149	0.882	0.029 (-0.037 – 0.096)	0.873	0.384	0.025 (-0.057 – 0.107)	0.611	0.543
TREM2	0.05 (-0.011 – 0.11)	1.627	0.106	0.05 (-0.014 – 0.114)	1.536	0.127	0.019 (-0.065 – 0.104)	0.456	0.649
YKL-40	-0.041 (-0.093 – 0.01)	-1.574	0.118	-0.003 (-0.061 – 0.054)	-0.116	0.908	0.083 (-0.018 – 0.184)	1.630	0.106
VEGF-A	0.023 (-0.039 – 0.085)	0.739	0.461	-0.021 (-0.101 – 0.058)	-0.527	0.600	-0.004 (-0.131 – 0.122)	-0.064	0.949
VEGF-B	0.031 (-0.025 – 0.087)	1.104	0.271	-0.047 (-0.1 – 0.006)	-1.776	0.079	0.05 (-0.023 – 0.124)	1.367	0.175
VEGF-C	0.034 (-0.016 – 0.084)	1.329	0.186	0.017 (-0.044 – 0.079)	0.556	0.579	-0.007 (-0.082 – 0.067)	-0.199	0.843
VEGF-D	0 (-0.037 – 0.038)	0.014	0.989	-0.019 (-0.08 – 0.041)	-0.638	0.524	-0.03 (-0.118 – 0.058)	-0.674	0.502
PGF	0.027 (-0.015 – 0.069)	1.266	0.208	-0.053 (-0.12 – 0.014)	-1.575	0.118	0.018 (-0.062 – 0.099)	0.452	0.652
NRGN	0.015 (-0.026 – 0.056)	0.719	0.473	-0.018 (-0.082 – 0.047)	-0.538	0.592	0.034 (-0.045 – 0.113)	0.844	0.401
NPTX2	-0.011 (-0.059 – 0.037)	-0.451	0.653	-0.012 (-0.042 – 0.018)	-0.808	0.420	0.106 (0.014 – 0.199)	2.282	**0.024**
SV2A	-0.014 (-0.062 – 0.035)	-0.561	0.576	0.032 (-0.02 – 0.083)	1.221	0.224	-0.01 (-0.09 – 0.07)	-0.245	0.807
SYT1	0.015 (-0.02 – 0.049)	0.854	0.395	0.003 (-0.053 – 0.059)	0.109	0.914	-0.022 (-0.072 – 0.029)	-0.848	0.398
14-3-3 ζ/δ	-0.015 (-0.06 – 0.03)	-0.670	0.504	-0.04 (-0.115 – 0.035)	-1.050	0.296	0.086 (-0.042 – 0.214)	1.333	0.186
NfL	0.001 (-0.041 – 0.043)	0.054	0.957	-0.109 (-0.174 – -0.044)	-3.316	**0.001***	-0.026 (-0.103 – 0.052)	-0.661	0.510
NGF	0.03 (0 – 0.061)	2.007	**0.047**	0.029 (-0.019 – 0.077)	1.198	0.233	-0.022 (-0.091 – 0.047)	-0.627	0.532

* $p_{FDR} < 0.05$; ** $p_{FDR} < 0.01$; *** $p_{FDR} < 0.001$

Supplementary table 7. Main cross-sectional effects of CSF proteins on AD signature atrophy controlling for temporal meta-ROI tau.

Brain resilience (BR); main cross-sectional effects on AD signature cortical thickness across all diagnostic groups controlling for temporal meta-ROI tau									
	A+ CU			A+ MCI			A+ AD		
Variable	Std β coefficient (CI)	t	p	Std β coefficient (CI)	t	p	Std β coefficient (CI)	t	p
GFAP	-0.05 (-0.325 – 0.225)	-0.362	0.718	-0.156 (-0.587 – 0.274)	-0.722	0.472	-0.146 (-0.556 – 0.264)	-0.708	0.481
GRN	-0.006 (-0.466 – 0.455)	-0.025	0.980	0.564 (0.102 – 1.026)	2.427	**0.017**	0.027 (-0.665 – 0.72)	0.078	0.938
ICAM-1	0.121 (-0.216 – 0.458)	0.711	0.479	0.106 (-0.158 – 0.371)	0.799	0.426	0.058 (-0.432 – 0.547)	0.235	0.815
IL-15	0.103 (-0.21 – 0.415)	0.651	0.516	-0.479 (-0.925 – -0.033)	-2.136	**0.036**	-0.085 (-0.47 – 0.301)	-0.436	0.664
TREM2	0.149 (-0.218 – 0.516)	0.804	0.423	-0.033 (-0.497 – 0.431)	-0.142	0.887	-0.326 (-0.74 – 0.087)	-1.566	0.121
YKL-40	0.193 (-0.136 – 0.521)	1.162	0.248	-0.048 (-0.466 – 0.369)	-0.231	0.818	-0.507 (-0.965 – -0.049)	-2.198	**0.030**
VEGF-A	0.033 (-0.41 – 0.476)	0.149	0.882	0.551 (0.016 – 1.087)	2.048	**0.044**	-0.937 (-1.502 – -0.373)	-3.296	**0.001***
VEGF-B	0.122 (-0.235 – 0.479)	0.677	0.500	-0.168 (-0.577 – 0.24)	-0.822	0.414	0.162 (-0.225 – 0.55)	0.832	0.408
VEGF-C	0.344 (0.002 – 0.687)	1.995	**0.049**	0.293 (-0.149 – 0.735)	1.320	0.191	-0.055 (-0.396 – 0.286)	-0.318	0.751
VEGF-D	-0.16 (-0.441 – 0.122)	-1.125	0.263	-0.163 (-0.537 – 0.21)	-0.870	0.387	-0.053 (-0.459 – 0.354)	-0.257	0.798
PGF	-0.082 (-0.369 – 0.204)	-0.570	0.570	-0.217 (-0.661 – 0.227)	-0.973	0.334	-0.176 (-0.559 – 0.206)	-0.915	0.363
NRGN	0.2 (-0.079 – 0.478)	1.424	0.158	0.353 (-0.08 – 0.787)	1.621	0.109	0.314 (-0.062 – 0.69)	1.659	0.100
NPTX2	0.098 (-0.231 – 0.427)	0.590	0.556	0.086 (-0.177 – 0.349)	0.652	0.516	0.488 (0.069 – 0.907)	2.314	**0.023**
SV2A	0 (-0.324 – 0.325)	0.003	0.998	0.443 (0.075 – 0.811)	2.394	**0.019**	-0.088 (-0.474 – 0.298)	-0.453	0.651
SYT1	0.043 (-0.225 – 0.312)	0.320	0.749	0.242 (-0.159 – 0.643)	1.201	0.233	0.019 (-0.237 – 0.274)	0.144	0.886
14-3-3 ζ/δ	0.071 (-0.245 – 0.387)	0.446	0.657	0.338 (-0.165 – 0.841)	1.340	0.185	0.036 (-0.582 – 0.654)	0.116	0.908
NfL	0.034 (-0.293 – 0.362)	0.208	0.836	-0.708 (-1.122 – -0.295)	-3.408	**0.001***	0.115 (-0.229 – 0.459)	0.664	0.508
NGF	0.037 (-0.175 – 0.249)	0.348	0.729	0.232 (-0.082 – 0.545)	1.471	0.145	-0.068 (-0.404 – 0.267)	-0.405	0.687

* $p_{FDR} < 0.05$; ** $p_{FDR} < 0.01$; *** $p_{FDR} < 0.001$

Supplementary table 8. Interactive effects of CSF proteins with whole brain tau on longitudinal whole brain cortical atrophy.

Brain resilience (BR); interaction effects with whole brain tau (Time × Tau × Variable β) on longitudinal whole brain cortical atrophy across all diagnostic groups									
	A+ CU			A+ MCI			A+ AD		
Variable	Std β coefficient (CI)	t	p	Std β coefficient (CI)	t	p	Std β coefficient (CI)	t	p
GFAP	-0.012 (-0.024 – 0.001)	-1.849	0.066	0 (-0.014 – 0.014)	-0.002	0.998	-0.008 (-0.021 – 0.005)	-1.272	0.206
GRN	-0.01 (-0.026 – 0.006)	-1.211	0.228	-0.007 (-0.02 – 0.007)	-0.964	0.337	-0.008 (-0.019 – 0.003)	-1.408	0.162
ICAM-1	-0.008 (-0.024 – 0.008)	-1.015	0.312	-0.002 (-0.016 – 0.012)	-0.238	0.813	-0.005 (-0.017 – 0.007)	-0.773	0.441
IL-15	-0.01 (-0.027 – 0.008)	-1.088	0.278	-0.006 (-0.02 – 0.008)	-0.797	0.427	-0.007 (-0.018 – 0.004)	-1.206	0.231
TREM2	0.002 (-0.018 – 0.021)	0.171	0.864	-0.001 (-0.015 – 0.013)	-0.167	0.868	-0.007 (-0.018 – 0.003)	-1.336	0.184
YKL-40	-0.01 (-0.031 – 0.011)	-0.934	0.352	-0.013 (-0.029 – 0.003)	-1.570	0.119	-0.009 (-0.024 – 0.005)	-1.292	0.199
VEGF-A	-0.01 (-0.026 – 0.005)	-1.296	0.197	-0.007 (-0.02 – 0.006)	-1.046	0.298	-0.01 (-0.02 – 0)	-1.897	0.061
VEGF-B	-0.008 (-0.027 – 0.012)	-0.780	0.437	-0.022 (-0.04 – -0.004)	-2.367	**0.020**	-0.006 (-0.015 – 0.003)	-1.313	0.192
VEGF-C	-0.012 (-0.031 – 0.007)	-1.232	0.220	-0.009 (-0.02 – 0.001)	-1.708	0.090	-0.01 (-0.019 – -0.001)	-2.256	**0.026**
VEGF-D	-0.011 (-0.025 – 0.003)	-1.595	0.113	-0.004 (-0.015 – 0.008)	-0.601	0.549	-0.011 (-0.022 – 0)	-1.908	0.059
PGF	-0.008 (-0.021 – 0.006)	-1.089	0.278	-0.001 (-0.013 – 0.011)	-0.156	0.876	-0.01 (-0.022 – 0.002)	-1.666	0.099
NRGN	-0.008 (-0.027 – 0.011)	-0.798	0.426	-0.018 (-0.033 – -0.004)	-2.500	**0.014**	0 (-0.01 – 0.01)	-0.057	0.955
NPTX2	-0.007 (-0.029 – 0.014)	-0.660	0.510	-0.011 (-0.023 – 0.001)	-1.778	0.078	-0.006 (-0.015 – 0.003)	-1.297	0.197
SV2A	-0.007 (-0.033 – 0.02)	-0.503	0.616	-0.018 (-0.038 – 0.002)	-1.738	0.085	0.003 (-0.008 – 0.015)	0.563	0.575
SYT1	-0.013 (-0.036 – 0.011)	-1.058	0.292	-0.023 (-0.042 – -0.004)	-2.415	**0.017**	-0.002 (-0.013 – 0.009)	-0.319	0.750
14-3-3 ζ/δ	-0.012 (-0.032 – 0.009)	-1.143	0.255	-0.017 (-0.035 – 0.001)	-1.902	0.060	-0.003 (-0.02 – 0.014)	-0.375	0.708
NfL	-0.009 (-0.024 – 0.005)	-1.240	0.217	-0.007 (-0.024 – 0.009)	-0.861	0.391	-0.011 (-0.024 – 0.002)	-1.678	0.096
NGF	0.015 (-0.008 – 0.038)	1.303	0.195	-0.009 (-0.027 – 0.009)	-1.018	0.311	-0.001 (-0.015 – 0.013)	-0.110	0.913

* $p_{FDR} < 0.05$; ** $p_{FDR} < 0.01$; *** $p_{FDR} < 0.001$

Supplementary table 9. Main longitudinal effects of CSF proteins on whole brain cortical atrophy controlling for whole brain tau.

Brain resilience (BR); main longitudinal effects (Time × Variable β) on whole brain cortical atrophy across all diagnostic groups controlling for whole brain tau									
	A+ CU			A+ MCI			A+ AD		
Variable	Std β coefficient (CI)	t	p	Std β coefficient (CI)	t	p	Std β coefficient (CI)	t	p
GFAP	0 (-0.038 – 0.038)	-0.013	0.990	0.002 (-0.055 – 0.059)	0.071	0.944	-0.099 (-0.207 – 0.01)	-1.806	0.074
GRN	-0.03 (-0.096 – 0.037)	-0.880	0.380	-0.015 (-0.076 – 0.047)	-0.467	0.641	0.026 (-0.148 – 0.199)	0.296	0.768
ICAM-1	-0.042 (-0.089 – 0.004)	-1.808	0.073	-0.001 (-0.04 – 0.039)	-0.027	0.978	-0.038 (-0.162 – 0.086)	-0.603	0.548
IL-15	0 (-0.042 – 0.042)	0.0120	0.991	-0.001 (-0.058 – 0.057)	-0.027	0.978	-0.002 (-0.1 – 0.096)	-0.038	0.969
TREM2	0.02 (-0.035 – 0.075)	0.724	0.470	-0.005 (-0.061 – 0.051)	-0.177	0.860	0.043 (-0.059 – 0.145)	0.831	0.408
YKL-40	-0.016 (-0.062 – 0.031)	-0.668	0.505	-0.025 (-0.074 – 0.025)	-0.976	0.331	0.073 (-0.05 – 0.197)	1.178	0.242
VEGF-A	-0.014 (-0.071 – 0.042)	-0.508	0.612	-0.044 (-0.112 – 0.024)	-1.287	0.201	0.036 (-0.12 – 0.193)	0.460	0.647
VEGF-B	0.012 (-0.039 – 0.062)	0.457	0.648	0.003 (-0.045 – 0.05)	0.118	0.907	0.039 (-0.05 – 0.128)	0.860	0.392
VEGF-C	0.029 (-0.015 – 0.072)	1.304	0.194	0.013 (-0.041 – 0.067)	0.479	0.633	0.019 (-0.071 – 0.109)	0.414	0.680
VEGF-D	-0.003 (-0.037 – 0.031)	-0.157	0.875	0.012 (-0.04 – 0.065)	0.464	0.643	-0.033 (-0.141 – 0.074)	-0.615	0.540
PGF	-0.009 (-0.047 – 0.029)	-0.488	0.626	0.008 (-0.051 – 0.066)	0.265	0.791	0.037 (-0.061 – 0.136)	0.750	0.455
NRGN	0.013 (-0.024 – 0.05)	0.692	0.490	-0.028 (-0.083 – 0.027)	-1.007	0.316	-0.036 (-0.132 – 0.06)	-0.747	0.457
NPTX2	0.009 (-0.035 – 0.053)	0.399	0.691	0.017 (-0.009 – 0.043)	1.319	0.190	0.07 (-0.044 – 0.185)	1.214	0.227
SV2A	-0.002 (-0.047 – 0.044)	-0.070	0.944	-0.002 (-0.048 – 0.043)	-0.094	0.925	-0.064 (-0.16 – 0.032)	-1.330	0.186
SYT1	0.008 (-0.024 – 0.039)	0.488	0.626	-0.028 (-0.076 – 0.02)	-1.156	0.250	-0.03 (-0.092 – 0.031)	-0.976	0.331
14-3-3 ζ/δ	0.008 (-0.033 – 0.049)	0.391	0.697	-0.06 (-0.12 – 0)	-1.975	0.051	-0.041 (-0.202 – 0.119)	-0.513	0.609
NfL	-0.007 (-0.045 – 0.03)	-0.385	0.701	-0.047 (-0.104 – 0.01)	-1.621	0.108	0.003 (-0.091 – 0.096)	0.059	0.953
NGF	0.017 (-0.01 – 0.045)	1.238	0.218	0.003 (-0.039 – 0.045)	0.150	0.881	-0.05 (-0.134 – 0.034)	-1.191	0.236

* $p_{FDR} < 0.05$; ** $p_{FDR} < 0.01$; *** $p_{FDR} < 0.001$

Supplementary table 10. Main cross-sectional effects of CSF proteins on whole brain cortical atrophy controlling for whole brain tau.

Brain resilience (BR); main cross-sectional effects on whole brain cortical thickness across all diagnostic groups controlling for whole brain tau									
	A+ CU			A+ MCI			A+ AD		
Variable	Std β coefficient (CI)	t	p	Std β coefficient (CI)	t	p	Std β coefficient (CI)	t	p
GFAP	-0.027 (-0.306 – 0.252)	-0.191	0.849	-0.134 (-0.44 – 0.172)	-0.871	0.386	-0.051 (-0.423 – 0.322)	-0.270	0.788
GRN	-0.537 (-0.977 – -0.097)	-2.422	**0.017**	0.153 (-0.191 – 0.496)	0.883	0.380	0.335 (-0.292 – 0.961)	1.061	0.291
ICAM-1	-0.125 (-0.456 – 0.207)	-0.747	0.457	0.083 (-0.107 – 0.273)	0.867	0.388	0.279 (-0.16 – 0.718)	1.260	0.211
IL-15	-0.137 (-0.447 – 0.174)	-0.874	0.384	-0.178 (-0.477 – 0.122)	-1.178	0.242	-0.13 (-0.481 – 0.221)	-0.735	0.464
TREM2	0.181 (-0.181 – 0.543)	0.994	0.323	-0.434 (-0.726 – -0.142)	-2.953	**0.004**	-0.096 (-0.473 – 0.28)	-0.508	0.613
YKL-40	0.067 (-0.261 – 0.396)	0.407	0.685	0.113 (-0.184 – 0.41)	0.758	0.450	-0.19 (-0.613 – 0.232)	-0.894	0.373
VEGF-A	0.085 (-0.354 – 0.523)	0.383	0.703	0.093 (-0.271 – 0.458)	0.508	0.612	-0.623 (-1.16 – -0.085)	-2.300	**0.024**
VEGF-B	0.041 (-0.308 – 0.39)	0.234	0.815	0.003 (-0.323 – 0.329)	0.021	0.983	0.091 (-0.255 – 0.436)	0.521	0.604
VEGF-C	0.216 (-0.141 – 0.573)	1.199	0.233	0.098 (-0.215 – 0.411)	0.623	0.535	-0.115 (-0.422 – 0.191)	-0.747	0.457
VEGF-D	-0.005 (-0.285 – 0.275)	-0.035	0.972	-0.027 (-0.277 – 0.222)	-0.218	0.828	0.136 (-0.233 – 0.505)	0.729	0.468
PGF	0.074 (-0.209 – 0.357)	0.517	0.607	0.047 (-0.249 – 0.342)	0.314	0.754	-0.027 (-0.373 – 0.319)	-0.154	0.878
NRGN	0.042 (-0.236 – 0.32)	0.301	0.764	0.018 (-0.282 – 0.318)	0.121	0.904	0.213 (-0.126 – 0.552)	1.247	0.215
NPTX2	0.173 (-0.147 – 0.494)	1.073	0.286	0.014 (-0.173 – 0.201)	0.146	0.885	0.244 (-0.142 – 0.63)	1.254	0.213
SV2A	0.049 (-0.266 – 0.363)	0.307	0.759	0.005 (-0.274 – 0.285)	0.038	0.970	-0.123 (-0.467 – 0.221)	-0.711	0.479
SYT1	0.05 (-0.216 – 0.315)	0.371	0.712	0.015 (-0.271 – 0.301)	0.107	0.915	0.066 (-0.164 – 0.296)	0.569	0.571
14-3-3 ζ/δ	-0.026 (-0.332 – 0.281)	-0.168	0.867	0.29 (-0.075 – 0.655)	1.584	0.118	0.037 (-0.522 – 0.595)	0.131	0.896
NfL	-0.115 (-0.434 – 0.204)	-0.716	0.476	0.026 (-0.3 – 0.352)	0.158	0.875	0.04 (-0.267 – 0.346)	0.256	0.798
NGF	0.093 (-0.116 – 0.301)	0.882	0.380	0.028 (-0.198 – 0.255)	0.249	0.804	-0.1 (-0.402 – 0.202)	-0.656	0.513

* $p_{FDR} < 0.05$; ** $p_{FDR} < 0.01$; *** $p_{FDR} < 0.001$

Supplementary table 11. Model performance metrics of the cognitive resilience analyses.

		A+ CU (mPACC5)						A+ MCI (MMSE)						A+ AD (MMSE)					
Variable		AIC	ΔAIC	R2	ΔR2	N (subj)	N (obs)	AIC	ΔAIC	R2	ΔR2	N (subj)	N (obs)	AIC	ΔAIC	R2	ΔR2	N (subj)	N (obs)
GFAP	Benchmark	738.98	0.00	33.75	0.00	106	337	1723.20	0.00	46.57	0.00	105	364	2039.27	0.00	40.06	0.00	138	375
	Main	742.59	-3.61	33.87	0.12	106	337	1726.31	-3.11	46.70	0.13	105	364	2041.10	-1.82	40.21	0.15	138	375
	Interaction	731.54	7.44	33.22	-0.53	106	337	1728.27	-5.08	47.29	0.72	105	364	2038.91	0.36	41.12	1.05	138	375
GRN	Benchmark	735.17	0.00	33.70	0.00	107	336	1765.57	0.00	45.87	0.00	107	373	2089.02	0.00	40.15	0.00	141	383
	Main	737.24	-2.07	33.70	0.01	107	336	1769.45	-3.88	45.87	-0.01	107	373	2092.33	-3.31	40.25	0.10	141	383
	Interaction	738.01	-2.85	33.08	-0.62	107	336	1772.39	-6.82	45.91	0.03	107	373	2091.44	-2.42	40.64	0.49	141	383
ICAM-1	Benchmark	744.39	0.00	33.73	0.00	108	341	1777.14	0.00	46.41	0.00	108	376	2092.81	0.00	40.50	0.00	141	384
	Main	746.84	-2.45	34.01	0.28	108	341	1780.92	-3.79	46.50	0.09	108	376	2096.14	-3.33	40.72	0.22	141	384
	Interaction	745.23	-0.84	33.41	-0.32	108	341	1784.83	-7.69	46.45	0.04	108	376	2096.96	-4.14	41.04	0.53	141	384
IL-15	Benchmark	724.30	0.00	35.59	0.00	105	333	1750.90	0.00	46.88	0.00	107	371	2063.80	0.00	41.86	0.00	140	380
	Main	726.93	-2.63	35.71	0.12	105	333	1750.07	0.83	47.73	0.84	107	371	2067.65	-3.84	41.91	0.05	140	380
	Interaction	722.72	1.58	35.87	0.28	105	333	1752.53	-1.64	47.69	0.80	107	371	2067.57	-3.77	42.24	0.38	140	380
TREM2	Benchmark	715.81	0.00	36.00	0.00	103	325	1734.96	0.00	46.95	0.00	106	368	2103.28	0.00	40.44	0.00	142	386
	Main	719.46	-3.65	36.29	0.29	103	325	1730.52	4.43	47.99	1.03	106	368	2101.11	2.17	42.05	1.62	142	386
	Interaction	718.37	-2.56	36.58	0.58	103	325	1730.97	3.99	49.23	2.28	106	368	2101.38	1.90	42.18	1.75	142	386
YKL-40	Benchmark	744.39	0.00	33.73	0.00	108	341	1777.14	0.00	46.41	0.00	108	376	2092.81	0.00	40.50	0.00	141	384
	Main	746.24	-1.86	35.09	1.36	108	341	1781.02	-3.89	46.47	0.05	108	376	2088.51	4.31	42.51	2.01	141	384
	Interaction	750.06	-5.67	35.34	1.61	108	341	1783.14	-6.00	46.41	0.00	108	376	2089.93	2.88	42.63	2.13	141	384
VEGF-A	Benchmark	732.07	0.00	35.59	0.00	106	336	1722.60	0.00	47.93	0.00	106	366	2103.28	0.00	40.44	0.00	142	386
	Main	734.17	-2.11	35.64	0.05	106	336	1725.63	-3.02	47.81	-0.11	106	366	2103.44	-0.16	40.81	0.37	142	386
	Interaction	724.38	7.69	35.37	-0.22	106	336	1728.96	-6.35	47.62	-0.30	106	366	2103.45	-0.18	40.82	0.39	142	386
VEGF-B	Benchmark	697.37	0.00	35.79	0.00	102	322	1579.82	0.00	45.93	0.00	96	332	1977.27	0.00	40.12	0.00	134	365
	Main	700.42	-3.04	35.95	0.16	102	322	1578.38	1.45	47.89	1.96	96	332	1979.50	-2.23	40.62	0.50	134	365
	Interaction	695.06	2.32	37.85	2.06	102	322	1582.14	-2.32	47.80	1.87	96	332	1982.67	-5.40	40.65	0.53	134	365
VEGF-C	Benchmark	732.99	0.00	33.98	0.00	106	334	1719.65	0.00	46.40	0.00	104	364	2083.32	0.00	42.09	0.00	141	383
	Main	731.50	1.49	34.28	0.30	106	334	1723.61	-3.97	46.44	0.04	104	364	2086.21	-2.89	42.23	0.14	141	383
	Interaction	732.46	0.53	34.16	0.17	106	334	1727.06	-7.41	46.57	0.17	104	364	2087.92	-4.59	42.55	0.46	141	383
VEGF-D	Benchmark	732.07	0.00	35.59	0.00	106	336	1722.60	0.00	47.93	0.00	106	366	2103.28	0.00	40.44	0.00	142	386
	Main	735.36	-3.29	36.29	0.70	106	336	1715.72	6.89	51.23	3.30	106	366	2103.13	0.15	40.67	0.24	142	386
	Interaction	718.66	13.40	38.47	2.88	106	336	1716.26	6.34	52.06	4.13	106	366	2105.36	-2.09	40.65	0.21	142	386
PGF	Benchmark	732.07	0.00	35.59	0.00	106	336	1722.60	0.00	47.93	0.00	106	366	2103.28	0.00	40.44	0.00	142	386
	Main	735.66	-3.60	35.83	0.24	106	336	1722.48	0.13	48.76	0.84	106	366	2106.77	-3.49	40.45	0.02	142	386
	Interaction	731.66	0.40	36.59	1.00	106	336	1726.31	-3.71	48.79	0.86	106	366	2105.90	-2.62	41.04	0.60	142	386
NRGN	Benchmark	744.39	0.00	33.73	0.00	108	341	1737.86	0.00	46.31	0.00	106	367	2089.45	0.00	40.44	0.00	141	383
	Main	746.11	-1.72	34.11	0.38	108	341	1739.30	-1.44	46.59	0.28	106	367	2091.63	-2.17	41.02	0.58	141	383
	Interaction	744.65	-0.26	33.48	-0.25	108	341	1741.91	-4.04	47.25	0.93	106	367	2094.68	-5.23	41.07	0.63	141	383
NPTX2	Benchmark	738.87	0.00	33.47	0.00	107	336	1765.37	0.00	46.09	0.00	107	373	2103.28	0.00	40.44	0.00	142	386
	Main	739.40	-0.53	34.83	1.35	107	336	1767.05	-1.68	46.45	0.36	107	373	2100.12	3.16	42.15	1.71	142	386
	Interaction	741.90	-3.03	35.39	1.91	107	336	1763.92	1.45	48.42	2.33	107	373	2100.28	3.00	42.30	1.86	142	386
SV2A	Benchmark	732.36	0.00	33.82	0.00	106	334	1736.25	0.00	46.56	0.00	105	367	2022.52	0.00	40.75	0.00	137	371
	Main	736.19	-3.83	33.90	0.07	106	334	1731.80	4.45	47.54	0.99	105	367	2025.70	-3.18	40.99	0.23	137	371
	Interaction	739.32	-6.96	33.19	-0.64	106	334	1735.29	0.96	47.56	1.01	105	367	2026.62	-4.09	41.02	0.26	137	371

SYT1	Benchmark	722.48	0.00	36.01	0.00	106	334	1765.14	0.00	46.15	0.00	107	373	2087.02	0.00	40.47	0.00	141	383
	Main	724.43	-1.95	36.40	0.39	106	334	1760.56	4.58	48.10	1.95	107	373	2089.91	-2.89	40.82	0.35	141	383
	Interaction	724.30	-1.82	35.48	-0.53	106	334	1762.41	2.73	48.48	2.33	107	373	2092.26	-5.25	40.74	0.27	141	383
14-3-3 ζ/δ	Benchmark	685.71	0.00	35.67	0.00	96	307	1560.69	0.00	46.92	0.00	90	328	1903.48	0.00	39.24	0.00	127	350
	Main	687.84	-2.13	36.55	0.88	96	307	1562.18	-1.49	47.28	0.36	90	328	1906.30	-2.82	39.67	0.43	127	350
	Interaction	683.29	2.42	36.52	0.85	96	307	1562.55	-1.86	48.11	1.19	90	328	1909.36	-5.88	39.68	0.44	127	350
NFL	Benchmark	741.70	0.00	33.60	0.00	107	339	1765.80	0.00	46.05	0.00	107	373	2049.76	0.00	39.55	0.00	138	375
	Main	744.38	-2.68	33.81	0.20	107	339	1751.34	14.46	54.06	8.01	107	373	2043.04	6.72	41.53	1.98	138	375
	Interaction	728.89	12.81	36.51	2.90	107	339	1755.12	10.68	54.05	8.00	107	373	2043.23	6.53	42.02	2.48	138	375
NGF	Benchmark	744.39	0.00	33.73	0.00	108	341	1759.48	0.00	46.46	0.00	107	372	2089.02	0.00	40.15	0.00	141	383
	Main	743.66	0.73	36.36	2.62	108	341	1758.66	0.82	47.61	1.15	107	372	2086.55	2.47	40.70	0.54	141	383
	Interaction	705.63	38.75	47.34	13.61	108	341	1761.29	-1.81	48.25	1.79	107	372	2088.62	0.41	40.92	0.77	141	383

Supplementary table 12. Results from cognitive resilience LASSO regressions.

	A+ CU (mPACC5; n=88)	A+ MCI (MMSE; n=80)	A+ AD (MMSE; n=117)
Age	-0.0061	-0.043	-0.030
Sex	-0.041	0.29	-0.20
Mean NPX	0.063	0.58	0.79
Tau	-1.1	-1.2	-0.99
GFAP	0	0	0
GRN	0	0	0
ICAM-1	-0.093	0	0
IL-15	0	0	0
TREM2	0.026	0	0
YKL-40	0.075	0	-0.47
VEGF-A	0.0038	0	0
VEGF-B	0	-0.14	0
VEGF-C	0.10	0	0
VEGF-D	0	0	0
PGF	0	0	0
NRGN	-0.046	0	0
NPTX2	0	0	0.11
SV2A	0	0	0
SYT1	0.053	0	0
NfL	0	-0.31	-0.010
NGF	-3.3	0	0
GFAP*tau	0	0	-0.062
GRN*tau	0	0	0
ICAM-1*tau	0	0	0
IL-15*tau	-0.048	0	0
TREM2*tau	0	0.056	-0.047
YKL-40*tau	0	0	0
VEGF-A*tau	0	0	0
VEGF-B*tau	0	0	0
VEGF-C*tau	0	0	0
VEGF-D*tau	-0.012	0	0
PGF*tau	0	0	-0.027
NRGN*tau	-0.022	0	0
NPTX2*tau	0	0	0
SV2A*tau	0	0	0

SYT1*tau	0	0	0
NfL*tau	-0.046	0	0
NGF*tau	2.93	0	0.52

Estimates from the LASSO regression in the cognitive resilience sample with the slopes of mPACC5 (A+ CU) or MMSE (A+ MCI and A+ AD) as outcome.

Supplementary table 13. Main longitudinal effects of CSF proteins on global cognition controlling for temporal meta-ROI tau.

Cognitive resilience (CR); main longitudinal effects (Time × Variable β) on global cognition across all diagnostic groups controlling for temporal meta-ROI tau									
	A+ CU (mPACC5)			A+ MCI (MMSE)			A+ AD (MMSE)		
Variable	Std β coefficient (CI)	t	p	Std β coefficient (CI)	t	p	Std β coefficient (CI)	t	p
GFAP	0.043 (-0.101 – 0.186)	0.590	0.557	0.025 (-0.336 – 0.387)	0.137	0.891	-0.33 (-0.768 – 0.108)	-1.493	0.138
GRN	0.097 (-0.138 – 0.332)	0.820	0.414	-0.032 (-0.467 – 0.404)	-0.143	0.886	-0.128 (-0.891 – 0.635)	-0.332	0.740
ICAM-1	-0.053 (-0.222 – 0.117)	-0.619	0.537	0.01 (-0.248 – 0.268)	0.075	0.940	-0.057 (-0.59 – 0.476)	-0.212	0.832
IL-15	-0.087 (-0.24 – 0.065)	-1.141	0.257	-0.37 (-0.781 – 0.04)	-1.789	0.077	-0.082 (-0.499 – 0.335)	-0.390	0.697
TREM2	0.039 (-0.155 – 0.233)	0.402	0.689	0.408 (0.021 – 0.795)	2.092	**0.039**	-0.485 (-0.981 – 0.012)	-1.933	0.056
YKL-40	0.074 (-0.092 – 0.241)	0.884	0.379	0.016 (-0.334 – 0.365)	0.088	0.930	-0.481 (-0.941 – -0.021)	-2.072	**0.041**
VEGF-A	0.006 (-0.211 – 0.223)	0.055	0.956	0.217 (-0.249 – 0.683)	0.925	0.357	-0.364 (-0.963 – 0.235)	-1.204	0.231
VEGF-B	-0.089 (-0.27 – 0.092)	-0.979	0.330	-0.406 (-0.757 – -0.055)	-2.305	**0.024**	0.251 (-0.127 – 0.63)	1.324	0.190
VEGF-C	0.209 (0.028 – 0.39)	2.295	**0.024**	-0.017 (-0.387 – 0.352)	-0.092	0.927	0.196 (-0.172 – 0.564)	1.055	0.294
VEGF-D	-0.021 (-0.162 – 0.121)	-0.289	0.773	-0.186 (-0.497 – 0.126)	-1.181	0.240	-0.403 (-0.797 – -0.009)	-2.029	**0.045**
PGF	-0.029 (-0.167 – 0.108)	-0.422	0.674	-0.164 (-0.509 – 0.18)	-0.945	0.347	-0.115 (-0.51 – 0.28)	-0.578	0.564
NRGN	-0.102 (-0.239 – 0.036)	-1.473	0.145	0.178 (-0.186 – 0.542)	0.970	0.334	0.242 (-0.152 – 0.635)	1.219	0.226
NPTX2	0.093 (-0.07 – 0.257)	1.135	0.260	0.007 (-0.22 – 0.233)	0.060	0.952	0.3 (-0.106 – 0.707)	1.464	0.146
SV2A	0.02 (-0.141 – 0.182)	0.249	0.804	-0.031 (-0.297 – 0.234)	-0.233	0.816	0.103 (-0.31 – 0.516)	0.496	0.621
SYT1	0.013 (-0.115 – 0.142)	0.209	0.835	0.133 (-0.144 – 0.411)	0.953	0.343	0.037 (-0.252 – 0.326)	0.254	0.800
14-3-3 ζ/δ	-0.059 (-0.204 – 0.087)	-0.808	0.422	0.126 (-0.297 – 0.55)	0.593	0.555	-0.017 (-0.485 – 0.452)	-0.071	0.944
NfL	-0.087 (-0.236 – 0.063)	-1.153	0.253	-0.69 (-1.067 – -0.312)	-3.618	**<0.001***	-0.56 (-0.942 – -0.179)	-2.915	**0.004**
NGF	0.09 (-0.019 – 0.199)	1.635	0.105	0.239 (-0.02 – 0.498)	1.832	0.070	0.418 (0.095 – 0.74)	2.564	**0.012**

* $p_{FDR} < 0.05$; ** $p_{FDR} < 0.01$; *** $p_{FDR} < 0.001$

Supplementary table 14. Main cross-sectional effects of CSF proteins on global cognition controlling for temporal meta-ROI tau.

Cognitive resilience (CR); main cross-sectional effects on global cognition across all diagnostic groups controlling for temporal meta-ROI tau									
	A+ CU (mPACC5)			A+ MCI (MMSE)			A+ AD (MMSE)		
Variable	Std β coefficient (CI)	t	p	Std β coefficient (CI)	t	p	Std β coefficient (CI)	t	p
GFAP	-0.021 (-0.326 – 0.285)	-0.134	0.894	-0.212 (-0.658 – 0.234)	-0.942	0.348	0.155 (-0.745 – 1.054)	0.340	0.734
GRN	-0.25 (-0.738 – 0.237)	-1.017	0.312	0.094 (-0.461 – 0.649)	0.336	0.738	0.603 (-0.884 – 2.09)	0.802	0.424
ICAM-1	-0.208 (-0.566 – 0.15)	-1.151	0.252	0.067 (-0.252 – 0.387)	0.417	0.677	0.439 (-0.626 – 1.504)	0.814	0.417
IL-15	0.022 (-0.292 – 0.335)	0.137	0.891	0.423 (-0.081 – 0.928)	1.665	0.099	0.045 (-0.77 – 0.86)	0.109	0.913
TREM2	0.088 (-0.292 – 0.468)	0.460	0.646	-0.609 (-1.091 – -0.126)	-2.499	**0.014**	-0.628 (-1.603 – 0.347)	-1.273	0.205
YKL-40	0.213 (-0.124 – 0.551)	1.251	0.214	-0.077 (-0.534 – 0.38)	-0.333	0.740	-0.842 (-1.822 – 0.138)	-1.699	0.092
VEGF-A	0.317 (-0.138 – 0.773)	1.380	0.170	-0.169 (-0.78 – 0.441)	-0.550	0.584	1.058 (-0.186 – 2.301)	1.682	0.095
VEGF-B	-0.02 (-0.414 – 0.374)	-0.099	0.921	0.248 (-0.225 – 0.72)	1.042	0.300	0.06 (-0.812 – 0.932)	0.135	0.893
VEGF-C	-0.081 (-0.447 – 0.286)	-0.438	0.662	-0.03 (-0.498 – 0.438)	-0.126	0.900	-0.062 (-0.812 – 0.689)	-0.162	0.871
VEGF-D	-0.122 (-0.419 – 0.174)	-0.817	0.415	-0.531 (-0.917 – -0.145)	-2.725	**0.007**	0.206 (-0.649 – 1.061)	0.477	0.634
PGF	-0.075 (-0.365 – 0.214)	-0.516	0.607	-0.332 (-0.772 – 0.108)	-1.495	0.138	-0.143 (-0.965 – 0.679)	-0.345	0.731
NRGN	0.028 (-0.27 – 0.325)	0.184	0.855	0.241 (-0.216 – 0.697)	1.044	0.299	0.193 (-0.631 – 1.016)	0.462	0.645
NPTX2	0.28 (-0.064 – 0.623)	1.614	0.110	0.223 (-0.073 – 0.52)	1.494	0.138	0.876 (0.026 – 1.725)	2.037	**0.043**
SV2A	0.061 (-0.271 – 0.393)	0.364	0.716	0.504 (0.164 – 0.844)	2.939	**0.004**	0.291 (-0.535 – 1.118)	0.697	0.487
SYT1	0.196 (-0.075 – 0.467)	1.437	0.154	0.47 (0.105 – 0.834)	2.558	**0.012**	0.31 (-0.31 – 0.929)	0.989	0.324
14-3-3 ζ/δ	-0.2 (-0.524 – 0.123)	-1.229	0.222	0.376 (-0.199 – 0.952)	1.299	0.197	-0.511 (-1.455 – 0.433)	-1.071	0.286
NfL	-0.021 (-0.344 – 0.301)	-0.131	0.896	-0.41 (-0.921 – 0.102)	-1.588	0.115	-0.545 (-1.363 – 0.272)	-1.320	0.189
NGF	0.182 (-0.042 – 0.406)	1.613	0.110	0.129 (-0.209 – 0.468)	0.755	0.452	-0.134 (-0.788 – 0.52)	-0.406	0.686

* $p_{FDR} < 0.05$; ** $p_{FDR} < 0.01$; *** $p_{FDR} < 0.001$

Supplementary table 15. Conditional effects (dependent on the interaction term with tau) on mPACC5 in the A+ CU group.

Cognitive resilience (CR); conditional main cross-sectional and longitudinal effects on mPACC5 in the amyloid positive CU group						
	Conditional main cross-sectional effect			Conditional main longitudinal effect		
Variable	Std β coefficient (CI)	t	p	Std β coefficient (CI)	t	p
GFAP	-0.008 (-0.321 – 0.306)	-0.049	0.961	0.099 (-0.037 – 0.235)	1.445	0.152
GRN						
ICAM-1	-0.202 (-0.58 – 0.176)	-1.061	0.291	0.003 (-0.171 – 0.176)	0.032	0.974
IL-15	0.055 (-0.269 – 0.38)	0.338	0.736	-0.032 (-0.185 – 0.12)	-0.422	0.674
TREM2	0.212 (-0.222 – 0.646)	0.97	0.335	0.126 (-0.081 – 0.334)	1.208	0.23
YKL-40						
VEGF-A	0.335 (-0.125 – 0.796)	1.445	0.151	0.077 (-0.137 – 0.291)	0.716	0.476
VEGF-B	0.155 (-0.281 – 0.592)	0.706	0.482	0.002 (-0.186 – 0.19)	0.022	0.983
VEGF-C						
VEGF-D	-0.036 (-0.356 – 0.284)	-0.223	0.824	0.097 (-0.044 – 0.238)	1.366	0.175
PGF	-0.047 (-0.343 – 0.25)	-0.311	0.756	0.025 (-0.116 – 0.167)	0.357	0.722
NRGN	0.023 (-0.283 – 0.33)	0.15	0.881	-0.08 (-0.214 – 0.055)	-1.177	0.243
NPTX2						
SV2A						
SYT1	0.201 (-0.097 – 0.499)	1.338	0.184	0.092 (-0.056 – 0.24)	1.235	0.22
14-3-3 ζ/δ	-0.181 (-0.509 – 0.147)	-1.095	0.277	-0.016 (-0.16 – 0.128)	-0.219	0.827
NfL	0.033 (-0.295 – 0.361)	0.202	0.841	-0.033 (-0.173 – 0.107)	-0.473	0.637
NGF	0.04 (-0.192 – 0.272)	0.341	0.734	-0.011 (-0.112 – 0.089)	-0.225	0.822

* $p_{FDR} < 0.05$; ** $p_{FDR} < 0.01$; *** $p_{FDR} < 0.001$

Supplementary table 16. Interactive effects of CSF proteins with whole brain tau on longitudinal global cognition.

Cognitive resilience (CR); interaction effects with whole brain tau (Time × Tau × Variable β) on longitudinal global cognition across all diagnostic groups												
	A+ CU (mPACC5)			A+ CU (MMSE)			A+ MCI (MMSE)			A+ AD (MMSE)		
Variable	Std β coefficient (CI)	t	p	Std β coefficient (CI)	t	p	Std β coefficient (CI)	t	p	Std β coefficient (CI)	t	p
GFAP	-0.113 (-0.168 – -0.057)	-4.026	**<0.001****	-0.121 (-0.181 – -0.06)	-3.977	**<0.001****	0.055 (-0.025 – 0.134)	1.361	0.176	-0.081 (-0.142 – -0.02)	-2.637	**0.009**
GRN	-0.068 (-0.15 – 0.014)	-1.641	0.104	-0.112 (-0.199 – -0.026)	-2.571	**0.012**	0.046 (-0.03 – 0.121)	1.193	0.235	-0.088 (-0.14 – -0.036)	-3.337	**0.001***
ICAM-1	-0.086 (-0.164 – -0.007)	-2.150	**0.033**	-0.113 (-0.189 – -0.037)	-2.942	**0.004***	0.02 (-0.062 – 0.102)	0.475	0.636	-0.079 (-0.129 – -0.028)	-3.062	**0.003***
IL-15	-0.07 (-0.141 – 0.001)	-1.945	0.054	-0.09 (-0.176 – -0.003)	-2.055	**0.042**	0.053 (-0.035 – 0.142)	1.203	0.231	-0.067 (-0.112 – -0.022)	-2.922	**0.004**
TREM2	-0.052 (-0.132 – 0.028)	-1.295	0.198	-0.055 (-0.155 – 0.045)	-1.096	0.275	0.082 (0.001 – 0.163)	2.010	**0.047**	-0.064 (-0.114 – -0.014)	-2.518	**0.013**
YKL-40	-0.018 (-0.097 – 0.062)	-0.444	0.658	-0.125 (-0.219 – -0.031)	-2.638	**0.009**	0.014 (-0.084 – 0.112)	0.279	0.781	-0.065 (-0.121 – -0.008)	-2.257	**0.026**
VEGF-A	-0.112 (-0.197 – -0.027)	-2.620	**0.010**	-0.081 (-0.172 – 0.009)	-1.778	0.078	0.031 (-0.043 – 0.105)	0.824	0.411	-0.06 (-0.103 – -0.018)	-2.817	**0.006**
VEGF-B	-0.053 (-0.143 – 0.037)	-1.157	0.250	-0.07 (-0.178 – 0.039)	-1.276	0.205	0.002 (-0.107 – 0.111)	0.035	0.972	-0.02 (-0.056 – 0.017)	-1.080	0.282
VEGF-C	-0.067 (-0.141 – 0.008)	-1.762	0.081	-0.006 (-0.095 – 0.082)	-0.144	0.886	0.026 (-0.045 – 0.097)	0.717	0.475	-0.053 (-0.1 – -0.006)	-2.233	**0.027**
VEGF-D	-0.1 (-0.165 – -0.035)	-3.037	**0.003***	-0.087 (-0.153 – -0.021)	-2.598	**0.011**	0.032 (-0.032 – 0.096)	0.982	0.328	-0.06 (-0.103 – -0.016)	-2.705	**0.008**
PGF	-0.071 (-0.145 – 0.002)	-1.918	0.057	-0.088 (-0.159 – -0.016)	-2.420	**0.017**	-0.001 (-0.075 – 0.073)	-0.036	0.971	-0.094 (-0.144 – -0.045)	-3.776	**<0.001***
NRGN	-0.076 (-0.149 – -0.002)	-2.027	**0.045**	0.038 (-0.054 – 0.13)	0.816	0.416	0.004 (-0.073 – 0.081)	0.107	0.915	-0.026 (-0.069 – 0.017)	-1.196	0.234
NPTX2	-0.031 (-0.117 – 0.054)	-0.726	0.469	-0.031 (-0.136 – 0.074)	-0.582	0.562	0.058 (-0.01 – 0.127)	1.693	0.093	-0.022 (-0.057 – 0.014)	-1.196	0.234
SV2A	-0.028 (-0.133 – 0.078)	-0.517	0.606	-0.049 (-0.175 – 0.077)	-0.773	0.441	0.025 (-0.063 – 0.113)	0.560	0.577	-0.051 (-0.098 – -0.003)	-2.104	**0.037**
SYT1	-0.073 (-0.178 – 0.032)	-1.371	0.173	-0.133 (-0.25 – -0.017)	-2.265	**0.026**	0.027 (-0.064 – 0.117)	0.578	0.564	-0.033 (-0.08 – 0.014)	-1.382	0.169
14-3-3 ζ/δ	-0.115 (-0.21 – -0.021)	-2.408	**0.017**	-0.188 (-0.282 – -0.093)	-3.936	**<0.001****	0.061 (-0.047 – 0.17)	1.123	0.264	-0.07 (-0.133 – -0.008)	-2.213	**0.028**
NfL	-0.126 (-0.191 – -0.062)	-3.887	**<0.001****	-0.156 (-0.222 – -0.089)	-4.629	**<0.001*****	0.001 (-0.096 – 0.097)	0.010	0.992	-0.078 (-0.133 – -0.022)	-2.765	**0.007**
NGF	0.176 (0.109 – 0.242)	5.241	**<0.001*****	0.109 (0.029 – 0.188)	2.707	**0.008**	-0.012 (-0.112 – 0.089)	-0.232	0.817	-0.039 (-0.098 – 0.019)	-1.339	0.183

* $p_{FDR} < 0.05$; ** $p_{FDR} < 0.01$; *** $p_{FDR} < 0.001$

Supplementary table 17. Main longitudinal effects of CSF proteins on global cognition controlling for whole brain tau.

Cognitive resilience (CR); main longitudinal effects (Time × Variable β) on global cognition across all diagnostic groups controlling for whole brain tau												
	A+ CU (mPACC5)			A+ CU (MMSE)			A+ MCI (MMSE)			A+ AD (MMSE)		
Variable	Std β coefficient (CI)	t	p	Std β coefficient (CI)	t	p	Std β coefficient (CI)	t	p	Std β coefficient (CI)	t	p
GFAP	-0.009 (-0.172 – 0.155)	-0.104	0.917	0.038 (-0.175 – 0.252)	0.356	0.722	0.086 (-0.289 – 0.461)	0.456	0.649	-0.285 (-0.746 – 0.175)	-1.227	0.222
GRN	0.123 (-0.146 – 0.391)	0.910	0.365	0.116 (-0.236 – 0.467)	0.654	0.515	0.04 (-0.406 – 0.486)	0.179	0.858	-0.046 (-0.841 – 0.749)	-0.113	0.910
ICAM-1	-0.06 (-0.255 – 0.136)	-0.608	0.545	-0.13 (-0.38 – 0.119)	-1.037	0.303	0.059 (-0.204 – 0.322)	0.444	0.658	-0.086 (-0.635 – 0.464)	-0.309	0.757
IL-15	-0.104 (-0.278 – 0.071)	-1.182	0.240	-0.037 (-0.268 – 0.195)	-0.313	0.755	-0.416 (-0.834 – 0.003)	-1.971	0.051	0.015 (-0.414 – 0.444)	0.070	0.944
TREM2	0.043 (-0.178 – 0.264)	0.383	0.702	0.16 (-0.126 – 0.447)	1.108	0.270	0.414 (0.016 – 0.812)	2.066	**0.041**	-0.514 (-1.025 – -0.003)	-1.991	**0.049**
YKL-40	0.071 (-0.119 – 0.261)	0.740	0.462	0.084 (-0.162 – 0.331)	0.676	0.501	-0.07 (-0.427 – 0.287)	-0.389	0.698	-0.476 (-0.953 – 0.001)	-1.977	0.051
VEGF-A	-0.032 (-0.281 – 0.217)	-0.259	0.796	0.246 (-0.077 – 0.569)	1.513	0.133	0.262 (-0.215 – 0.739)	1.090	0.278	-0.487 (-1.108 – 0.133)	-1.558	0.122
VEGF-B	-0.06 (-0.272 – 0.152)	-0.565	0.574	-0.032 (-0.311 – 0.247)	-0.225	0.822	-0.427 (-0.786 – -0.069)	-2.373	**0.020**	0.312 (-0.08 – 0.704)	1.586	0.117
VEGF-C	0.203 (-0.007 – 0.413)	1.923	0.058	0.473 (0.214 – 0.732)	3.628	**<0.001****	0.001 (-0.378 – 0.379)	0.004	0.997	0.193 (-0.188 – 0.573)	1.001	0.319
VEGF-D	-0.064 (-0.226 – 0.098)	-0.782	0.436	-0.02 (-0.228 – 0.188)	-0.193	0.847	-0.211 (-0.531 – 0.108)	-1.310	0.193	-0.486 (-0.893 – -0.079)	-2.370	**0.020**
PGF	-0.045 (-0.203 – 0.113)	-0.565	0.573	-0.082 (-0.289 – 0.126)	-0.782	0.436	-0.166 (-0.519 – 0.188)	-0.929	0.355	-0.19 (-0.599 – 0.218)	-0.922	0.358
NRGN	-0.157 (-0.313 – -0.002)	-2.012	**0.048**	-0.071 (-0.276 – 0.134)	-0.69	0.492	0.013 (-0.353 – 0.38)	0.072	0.943	0.269 (-0.137 – 0.675)	1.314	0.192
NPTX2	0.141 (-0.046 – 0.327)	1.504	0.137	0.156 (-0.086 – 0.398)	1.279	0.204	-0.024 (-0.256 – 0.207)	-0.209	0.835	0.284 (-0.141 – 0.709)	1.325	0.188
SV2A	0.022 (-0.162 – 0.207)	0.242	0.810	0.048 (-0.193 – 0.289)	0.398	0.692	-0.059 (-0.332 – 0.214)	-0.429	0.669	0.191 (-0.232 – 0.615)	0.894	0.373
SYT1	0.011 (-0.14 – 0.161)	0.141	0.889	0 (-0.196 – 0.196)	0.000	1.000	0.129 (-0.156 – 0.415)	0.899	0.371	0.025 (-0.275 – 0.324)	0.165	0.869
14-3-3 ζ/δ	-0.109 (-0.278 – 0.059)	-1.295	0.200	-0.077 (-0.3 – 0.147)	-0.682	0.497	0.009 (-0.415 – 0.434)	0.044	0.965	-0.046 (-0.532 – 0.44)	-0.187	0.852
NfL	-0.125 (-0.296 – 0.047)	-1.450	0.151	-0.046 (-0.272 – 0.18)	-0.403	0.688	-0.753 (-1.132 – -0.373)	-3.934	**<0.001****	-0.517 (-0.917 – -0.117)	-2.564	**0.012**
NGF	0.116 (-0.008 – 0.239)	1.855	0.067	0.197 (0.041 – 0.353)	2.501	**0.014**	0.284 (0.022 – 0.547)	2.146	**0.034**	0.374 (0.041 – 0.708)	2.223	**0.028**

* $p_{FDR} < 0.05$; ** $p_{FDR} < 0.01$; *** $p_{FDR} < 0.001$

Supplementary table 18. Main cross-sectional effects of CSF proteins on global cognition controlling for whole brain tau.

Cognitive resilience (CR); main cross-sectional effects on global cognition across all diagnostic groups controlling for whole brain tau												
	A+ CU (mPACC5)			A+ CU (MMSE)			A+ MCI (MMSE)			A+ AD (MMSE)		
Variable	Std β coefficient (CI)	t	p	Std β coefficient (CI)	t	p	Std β coefficient (CI)	t	p	Std β coefficient (CI)	t	p
GFAP	-0.058 (-0.365 – 0.25)	-0.371	0.711	-0.011 (-0.358 – 0.335)	-0.066	0.948	-0.161 (-0.612 – 0.291)	-0.705	0.482	0.306 (-0.586 – 1.198)	0.679	0.498
GRN	-0.244 (-0.739 – 0.251)	-0.976	0.331	-0.105 (-0.679 – 0.469)	-0.361	0.719	0.17 (-0.38 – 0.719)	0.611	0.542	0.93 (-0.546 – 2.406)	1.246	0.215
ICAM-1	-0.239 (-0.602 – 0.123)	-1.307	0.194	-0.327 (-0.738 – 0.084)	-1.574	0.118	0.101 (-0.217 – 0.418)	0.628	0.531	0.464 (-0.584 – 1.512)	0.875	0.383
IL-15	0.013 (-0.306 – 0.332)	0.080	0.936	0.141 (-0.228 – 0.51)	0.758	0.450	0.426 (-0.075 – 0.927)	1.687	0.094	0.214 (-0.586 – 1.014)	0.529	0.597
TREM2	0.065 (-0.322 – 0.453)	0.335	0.738	-0.232 (-0.674 – 0.209)	-1.042	0.300	-0.621 (-1.101 – -0.142)	-2.568	**0.012**	-0.61 (-1.571 – 0.351)	-1.255	0.212
YKL-40	0.197 (-0.147 – 0.54)	1.135	0.259	0.108 (-0.282 – 0.497)	0.548	0.585	-0.136 (-0.59 – 0.318)	-0.592	0.555	-0.848 (-1.812 – 0.117)	-1.738	0.084
VEGF-A	0.314 (-0.15 – 0.778)	1.342	0.182	-0.188 (-0.723 – 0.346)	-0.697	0.487	-0.164 (-0.77 – 0.442)	-0.537	0.592	0.857 (-0.379 – 2.093)	1.371	0.173
VEGF-B	0.026 (-0.372 – 0.424)	0.129	0.898	0.149 (-0.308 – 0.606)	0.644	0.520	0.254 (-0.216 – 0.725)	1.075	0.286	0.085 (-0.77 – 0.94)	0.197	0.845
VEGF-C	-0.067 (-0.442 – 0.307)	-0.356	0.723	-0.334 (-0.758 – 0.089)	-1.562	0.121	-0.017 (-0.481 – 0.447)	-0.072	0.943	-0.07 (-0.809 – 0.668)	-0.189	0.851
VEGF-D	-0.125 (-0.428 – 0.177)	-0.821	0.414	-0.268 (-0.611 – 0.076)	-1.543	0.125	-0.546 (-0.929 – -0.162)	-2.82	**0.006**	0.078 (-0.765 – 0.921)	0.183	0.855
PGF	-0.1 (-0.395 – 0.194)	-0.677	0.500	-0.122 (-0.461 – 0.218)	-0.708	0.480	-0.338 (-0.774 – 0.098)	-1.535	0.128	-0.248 (-1.057 – 0.562)	-0.605	0.546
NRGN	-0.019 (-0.318 – 0.28)	-0.125	0.901	-0.052 (-0.394 – 0.289)	-0.302	0.763	0.152 (-0.295 – 0.6)	0.676	0.500	0.273 (-0.537 – 1.084)	0.666	0.506
NPTX2	0.326 (-0.019 – 0.67)	1.876	0.063	0.267 (-0.132 – 0.666)	1.323	0.188	0.205 (-0.089 – 0.499)	1.381	0.170	0.746 (-0.104 – 1.595)	1.736	0.085
SV2A	0.065 (-0.272 – 0.402)	0.380	0.704	0.181 (-0.205 – 0.567)	0.927	0.356	0.486 (0.146 – 0.826)	2.833	**0.006**	0.383 (-0.425 – 1.192)	0.938	0.350
SYT1	0.201 (-0.075 – 0.476)	1.444	0.152	0.17 (-0.153 – 0.492)	1.042	0.300	0.463 (0.099 – 0.826)	2.528	**0.013**	0.272 (-0.34 – 0.883)	0.879	0.381
14-3-3 ζ/δ	-0.238 (-0.564 – 0.088)	-1.449	0.151	-0.219 (-0.598 – 0.159)	-1.149	0.253	0.324 (-0.23 – 0.879)	1.162	0.248	-0.401 (-1.334 – 0.532)	-0.851	0.396
NfL	-0.058 (-0.383 – 0.267)	-0.352	0.726	-0.18 (-0.554 – 0.193)	-0.956	0.341	-0.421 (-0.928 – 0.087)	-1.644	0.103	-0.428 (-1.236 – 0.38)	-1.048	0.296
NGF	0.182 (-0.045 – 0.409)	1.592	0.114	0.212 (-0.043 – 0.468)	1.647	0.102	0.163 (-0.171 – 0.497)	0.968	0.335	-0.242 (-0.886 – 0.402)	-0.743	0.459

* $p_{FDR} < 0.05$; ** $p_{FDR} < 0.01$; *** $p_{FDR} < 0.001$

Supplementary table 19. Interactive effects of CSF proteins with temporal meta-ROI tau on longitudinal ADAS-Cog immediate recall.

Cognitive resilience (CR); interaction effects with temporal meta-ROI tau (Time × Tau × Variable β) on longitudinal ADAS-Cog immediate recall across all diagnostic groups									
	A+ CU			A+ MCI			A+ AD		
Variable	Std β coefficient (CI)	t	p	Std β coefficient (CI)	t	p	Std β coefficient (CI)	t	p
GFAP	0.008 (-0.018 – 0.034)	0.603	0.548	-0.011 (-0.032 – 0.01)	-1.066	0.288	-0.001 (-0.014 – 0.012)	-0.111	0.912
GRN	0.001 (-0.036 – 0.037)	0.032	0.975	-0.011 (-0.03 – 0.008)	-1.147	0.254	0.001 (-0.011 – 0.014)	0.221	0.825
ICAM-1	-0.01 (-0.044 – 0.025)	-0.547	0.585	-0.02 (-0.039 – 0)	-1.993	**0.048**	0.005 (-0.007 – 0.017)	0.830	0.408
IL-15	-0.006 (-0.041 – 0.029)	-0.346	0.730	-0.007 (-0.028 – 0.015)	-0.615	0.540	0.001 (-0.009 – 0.011)	0.212	0.832
TREM2	-0.001 (-0.034 – 0.033)	-0.051	0.960	-0.004 (-0.021 – 0.013)	-0.451	0.653	0 (-0.012 – 0.012)	0.030	0.976
YKL-40	0.014 (-0.021 – 0.049)	0.792	0.430	-0.006 (-0.028 – 0.016)	-0.567	0.572	0 (-0.012 – 0.012)	0.057	0.955
VEGF-A	0.012 (-0.022 – 0.046)	0.683	0.496	-0.014 (-0.034 – 0.005)	-1.482	0.141	-0.001 (-0.013 – 0.012)	-0.113	0.910
VEGF-B	-0.009 (-0.051 – 0.034)	-0.403	0.688	0.003 (-0.019 – 0.025)	0.270	0.788	-0.002 (-0.015 – 0.012)	-0.232	0.817
VEGF-C	0.01 (-0.023 – 0.043)	0.608	0.544	-0.015 (-0.032 – 0.002)	-1.798	0.075	-0.002 (-0.012 – 0.008)	-0.427	0.670
VEGF-D	-0.004 (-0.03 – 0.022)	-0.275	0.784	-0.013 (-0.029 – 0.003)	-1.627	0.106	-0.001 (-0.013 – 0.011)	-0.109	0.913
PGF	-0.005 (-0.035 – 0.026)	-0.299	0.766	-0.01 (-0.026 – 0.006)	-1.227	0.222	-0.006 (-0.019 – 0.008)	-0.844	0.400
NRGN	0.028 (-0.006 – 0.062)	1.638	0.104	-0.02 (-0.04 – -0.001)	-2.064	**0.042**	0.003 (-0.007 – 0.013)	0.574	0.568
NPTX2	0.028 (-0.008 – 0.064)	1.527	0.130	-0.001 (-0.017 – 0.015)	-0.110	0.912	0.001 (-0.014 – 0.017)	0.166	0.868
SV2A	0.031 (-0.012 – 0.075)	1.418	0.159	-0.015 (-0.036 – 0.005)	-1.466	0.146	0.004 (-0.009 – 0.017)	0.600	0.550
SYT1	0.003 (-0.042 – 0.049)	0.144	0.886	-0.022 (-0.044 – 0)	-1.998	**0.048**	0.001 (-0.013 – 0.015)	0.169	0.866
14-3-3 ζ/δ	-0.011 (-0.054 – 0.032)	-0.508	0.613	-0.007 (-0.032 – 0.018)	-0.585	0.560	0.009 (-0.006 – 0.025)	1.186	0.238
NfL	-0.016 (-0.044 – 0.012)	-1.135	0.258	-0.009 (-0.032 – 0.014)	-0.781	0.436	0 (-0.011 – 0.011)	0.007	0.994
NGF	0.022 (0 – 0.044)	1.948	0.053	0.004 (-0.014 – 0.022)	0.434	0.665	0.004 (-0.01 – 0.018)	0.530	0.597

* $p_{FDR} < 0.05$; ** $p_{FDR} < 0.01$; *** $p_{FDR} < 0.001$

Supplementary table 20. Main longitudinal effects of CSF proteins on ADAS-Cog immediate recall controlling for temporal meta-ROI tau.

Cognitive resilience (CR); main longitudinal effects (Time × Variable β) on ADAS-Cog immediate recall across all diagnostic groups controlling for temporal meta-ROI tau									
	A+ CU			A+ MCI			A+ AD		
Variable	Std β coefficient (CI)	t	p	Std β coefficient (CI)	t	p	Std β coefficient (CI)	t	p
GFAP	0.016 (-0.08 – 0.111)	0.322	0.748	0.022 (-0.101 – 0.145)	0.356	0.722	-0.052 (-0.204 – 0.1)	-0.680	0.498
GRN	0.117 (-0.041 – 0.275)	1.467	0.144	0.012 (-0.132 – 0.156)	0.171	0.865	-0.053 (-0.297 – 0.191)	-0.429	0.669
ICAM-1	0.023 (-0.093 – 0.138)	0.390	0.698	-0.054 (-0.144 – 0.037)	-1.17	0.244	0.022 (-0.152 – 0.197)	0.253	0.801
IL-15	-0.098 (-0.201 – 0.004)	-1.910	0.059	-0.052 (-0.187 – 0.083)	-0.768	0.445	0.041 (-0.089 – 0.172)	0.628	0.532
TREM2	0.039 (-0.096 – 0.174)	0.573	0.568	0.136 (0.016 – 0.257)	2.248	**0.027**	-0.051 (-0.188 – 0.086)	-0.749	0.457
YKL-40	0.069 (-0.043 – 0.181)	1.220	0.225	0.019 (-0.097 – 0.135)	0.320	0.750	-0.142 (-0.296 – 0.011)	-1.842	0.069
VEGF-A	-0.005 (-0.153 – 0.143)	-0.068	0.946	0.146 (-0.007 – 0.298)	1.903	0.060	-0.111 (-0.297 – 0.076)	-1.179	0.242
VEGF-B	-0.025 (-0.156 – 0.107)	-0.372	0.711	-0.068 (-0.176 – 0.04)	-1.26	0.212	-0.082 (-0.235 – 0.071)	-1.061	0.291
VEGF-C	0.033 (-0.093 – 0.16)	0.521	0.603	0.068 (-0.052 – 0.187)	1.126	0.263	-0.008 (-0.129 – 0.113)	-0.126	0.900
VEGF-D	-0.036 (-0.129 – 0.058)	-0.761	0.449	0.084 (-0.022 – 0.189)	1.573	0.119	0.108 (-0.016 – 0.231)	1.738	0.086
PGF	-0.067 (-0.158 – 0.024)	-1.459	0.149	0.018 (-0.098 – 0.135)	0.314	0.754	0.018 (-0.105 – 0.142)	0.297	0.767
NRGN	-0.029 (-0.12 – 0.063)	-0.619	0.537	0.113 (-0.008 – 0.233)	1.861	0.066	-0.11 (-0.235 – 0.014)	-1.767	0.082
NPTX2	0.044 (-0.064 – 0.152)	0.804	0.424	-0.011 (-0.083 – 0.06)	-0.319	0.751	0.067 (-0.07 – 0.203)	0.966	0.336
SV2A	0.084 (-0.024 – 0.192)	1.543	0.126	-0.025 (-0.109 – 0.059)	-0.595	0.554	0.033 (-0.101 – 0.167)	0.484	0.629
SYT1	-0.062 (-0.139 – 0.015)	-1.616	0.112	-0.037 (-0.124 – 0.05)	-0.839	0.405	-0.09 (-0.173 – -0.008)	-2.179	**0.032**
14-3-3 ζ/δ	-0.065 (-0.157 – 0.027)	-1.423	0.160	-0.032 (-0.165 – 0.102)	-0.473	0.638	-0.128 (-0.263 – 0.006)	-1.906	0.061
NfL	-0.042 (-0.148 – 0.065)	-0.778	0.438	-0.129 (-0.256 – -0.002)	-2.018	**0.047**	-0.055 (-0.185 – 0.074)	-0.848	0.398
NGF	0.034 (-0.04 – 0.108)	0.905	0.368	0.05 (-0.033 – 0.133)	1.194	0.236	-0.005 (-0.11 – 0.1)	-0.096	0.924

* $p_{FDR} < 0.05$; ** $p_{FDR} < 0.01$; *** $p_{FDR} < 0.001$

Supplementary table 21. Main cross-sectional effects of CSF proteins on ADAS-Cog immediate recall controlling for temporal meta-ROI tau.

Cognitive resilience (CR); main cross-sectional effects on ADAS-Cog immediate recall across all diagnostic groups controlling for temporal meta-ROI tau									
	A+ CU			A+ MCI			A+ AD		
Variable	Std β coefficient (CI)	t	p	Std β coefficient (CI)	t	p	Std β coefficient (CI)	t	p
GFAP	0.025 (-0.247 – 0.296)	0.180	0.858	-0.045 (-0.295 – 0.206)	-0.353	0.725	-0.072 (-0.368 – 0.224)	-0.481	0.631
GRN	0.068 (-0.382 – 0.517)	0.298	0.766	0.123 (-0.182 – 0.428)	0.799	0.426	0.442 (-0.055 – 0.939)	1.763	0.081
ICAM-1	-0.319 (-0.643 – 0.006)	-1.944	0.054	0.12 (-0.054 – 0.294)	1.369	0.174	0.138 (-0.214 – 0.49)	0.777	0.439
IL-15	0.036 (-0.252 – 0.324)	0.250	0.803	-0.11 (-0.393 – 0.173)	-0.772	0.442	-0.089 (-0.367 – 0.189)	-0.635	0.527
TREM2	0.189 (-0.158 – 0.536)	1.080	0.283	-0.328 (-0.594 – -0.062)	-2.448	**0.016**	0.136 (-0.181 – 0.453)	0.849	0.398
YKL-40	-0.06 (-0.363 – 0.242)	-0.395	0.693	-0.134 (-0.387 – 0.119)	-1.048	0.297	-0.346 (-0.676 – -0.017)	-2.085	**0.039**
VEGF-A	0.543 (0.139 – 0.947)	2.664	**0.009**	-0.059 (-0.4 – 0.282)	-0.345	0.731	0.181 (-0.231 – 0.592)	0.871	0.385
VEGF-B	-0.118 (-0.468 – 0.231)	-0.671	0.504	0.175 (-0.082 – 0.433)	1.352	0.180	-0.128 (-0.409 – 0.153)	-0.905	0.368
VEGF-C	0.089 (-0.241 – 0.418)	0.534	0.594	0.108 (-0.152 – 0.368)	0.827	0.410	0.006 (-0.249 – 0.26)	0.043	0.966
VEGF-D	0.154 (-0.117 – 0.424)	1.126	0.263	-0.085 (-0.309 – 0.138)	-0.757	0.450	-0.215 (-0.484 – 0.053)	-1.591	0.115
PGF	-0.023 (-0.288 – 0.242)	-0.169	0.866	-0.26 (-0.506 – -0.013)	-2.09	**0.039**	-0.12 (-0.391 – 0.15)	-0.881	0.380
NRGN	0.081 (-0.189 – 0.351)	0.596	0.552	0.189 (-0.058 – 0.436)	1.515	0.133	0.08 (-0.187 – 0.347)	0.592	0.555
NPTX2	-0.008 (-0.325 – 0.309)	-0.049	0.961	0.13 (-0.035 – 0.295)	1.564	0.121	0.211 (-0.077 – 0.5)	1.450	0.150
SV2A	0.022 (-0.282 – 0.326)	0.142	0.888	0.187 (-0.003 – 0.378)	1.955	0.053	0.211 (-0.074 – 0.496)	1.470	0.144
SYT1	0.338 (0.084 – 0.593)	2.637	**0.010**	0.07 (-0.137 – 0.278)	0.674	0.502	0.225 (0.037 – 0.412)	2.379	**0.019**
14-3-3 ζ/δ	-0.097 (-0.384 – 0.19)	-0.67	0.505	-0.032 (-0.343 – 0.279)	-0.205	0.838	0.086 (-0.233 – 0.406)	0.535	0.594
NfL	-0.009 (-0.308 – 0.291)	-0.057	0.954	-0.387 (-0.66 – -0.114)	-2.814	**0.006**	-0.363 (-0.631 – -0.095)	-2.684	**0.008**
NGF	0.026 (-0.179 – 0.231)	0.251	0.802	-0.131 (-0.314 – 0.052)	-1.422	0.158	0.173 (-0.048 – 0.394)	1.551	0.124

* $p_{FDR} < 0.05$; ** $p_{FDR} < 0.01$; *** $p_{FDR} < 0.001$

Supplementary table 22. Interactive effects of CSF proteins with temporal meta-ROI tau on longitudinal Trailmaking test A.

Cognitive resilience (CR); interaction effects with temporal meta-ROI tau (Time × Tau × Variable β) on longitudinal TMTA across all diagnostic groups									
	A+ CU			A+ MCI			A+ AD		
Variable	Std β coefficient (CI)	t	p	Std β coefficient (CI)	t	p	Std β coefficient (CI)	t	p
GFAP	-0.115 (-0.171 – -0.06)	-4.072	**<0.001*** **	-0.055 (-0.128 – 0.018)	-1.475	0.141	-0.039 (-0.155 – 0.076)	-0.675	0.501
GRN	-0.055 (-0.133 – 0.023)	-1.389	0.166	-0.063 (-0.122 – -0.003)	-2.066	**0.040**	-0.068 (-0.173 – 0.038)	-1.270	0.206
ICAM-1	-0.081 (-0.146 – -0.015)	-2.411	**0.017**	0.014 (-0.051 – 0.08)	0.428	0.669	-0.039 (-0.139 – 0.061)	-0.765	0.445
IL-15	-0.102 (-0.179 – -0.026)	-2.626	**0.009**	-0.102 (-0.174 – -0.03)	-2.773	**0.006**	-0.023 (-0.114 – 0.068)	-0.498	0.619
TREM2	-0.067 (-0.143 – 0.008)	-1.754	0.081	-0.015 (-0.071 – 0.04)	-0.539	0.590	-0.075 (-0.18 – 0.03)	-1.409	0.160
YKL-40	0.015 (-0.061 – 0.09)	0.381	0.703	0.003 (-0.069 – 0.075)	0.083	0.934	0.007 (-0.098 – 0.112)	0.133	0.894
VEGF-A	-0.147 (-0.224 – -0.071)	-3.793	**<0.001** **	-0.059 (-0.115 – -0.002)	-2.039	**0.042**	-0.038 (-0.138 – 0.063)	-0.738	0.461
VEGF-B	-0.173 (-0.267 – -0.078)	-3.591	**<0.001** **	-0.058 (-0.134 – 0.017)	-1.516	0.131	-0.035 (-0.127 – 0.057)	-0.758	0.449
VEGF-C	-0.024 (-0.102 – 0.055)	-0.593	0.554	-0.058 (-0.118 – 0.002)	-1.904	0.058	-0.058 (-0.146 – 0.03)	-1.294	0.197
VEGF-D	-0.14 (-0.194 – -0.086)	-5.105	**<0.001*** **	-0.019 (-0.069 – 0.031)	-0.763	0.446	-0.046 (-0.142 – 0.051)	-0.935	0.351
PGF	-0.099 (-0.163 – -0.034)	-3.022	**0.003***	0.039 (-0.015 – 0.093)	1.419	0.157	0.04 (-0.08 – 0.159)	0.652	0.515
NRGN	-0.146 (-0.225 – -0.068)	-3.681	**<0.001** **	0.011 (-0.061 – 0.083)	0.298	0.766	-0.01 (-0.093 – 0.073)	-0.231	0.818
NPTX2	-0.008 (-0.092 – 0.077)	-0.179	0.858	0.024 (-0.03 – 0.077)	0.873	0.383	-0.007 (-0.103 – 0.089)	-0.143	0.886
SV2A	-0.032 (-0.137 – 0.073)	-0.600	0.549	-0.133 (-0.211 – -0.055)	-3.369	**0.001***	-0.07 (-0.178 – 0.037)	-1.289	0.199
SYT1	-0.078 (-0.177 – 0.022)	-1.542	0.124	-0.007 (-0.093 – 0.08)	-0.151	0.880	-0.04 (-0.152 – 0.072)	-0.697	0.487
14-3-3 ζ/δ	-0.119 (-0.211 – -0.026)	-2.529	**0.012**	-0.029 (-0.12 – 0.062)	-0.631	0.528	-0.039 (-0.18 – 0.101)	-0.552	0.581
NfL	-0.138 (-0.194 – -0.082)	-4.814	**<0.001*** **	-0.058 (-0.134 – 0.018)	-1.496	0.136	0.004 (-0.091 – 0.099)	0.085	0.933
NGF	0.227 (0.177 – 0.277)	8.929	**<0.001*** **	0.038 (-0.029 – 0.105)	1.121	0.263	0.059 (-0.055 – 0.173)	1.020	0.309

* $p_{FDR} < 0.05$; ** $p_{FDR} < 0.01$; *** $p_{FDR} < 0.001$

Supplementary table 23. Main longitudinal effects of CSF proteins on Trailmaking test A controlling for temporal meta-ROI tau.

Cognitive resilience (CR); main longitudinal effects (Time × Variable β) on TMTA across all diagnostic groups controlling for temporal meta-ROI tau									
	A+ CU			A+ MCI			A+ AD		
Variable	Std β coefficient (CI)	t	p	Std β coefficient (CI)	t	p	Std β coefficient (CI)	t	p
GFAP	-0.031 (-0.249 – 0.187)	-0.279	0.780	-0.273 (-0.702 – 0.155)	-1.255	0.210	-1.369 (-2.644 – -0.093)	-2.116	**0.036**
GRN	-0.01 (-0.372 – 0.352)	-0.054	0.957	-0.389 (-0.905 – 0.128)	-1.482	0.139	-2.027 (-4.106 – 0.052)	-1.922	0.056
ICAM-1	-0.154 (-0.406 – 0.097)	-1.207	0.229	-0.063 (-0.399 – 0.274)	-0.365	0.715	-0.792 (-2.296 – 0.712)	-1.038	0.300
IL-15	-0.029 (-0.263 – 0.206)	-0.240	0.811	-0.503 (-0.966 – -0.039)	-2.135	**0.034**	0.693 (-0.461 – 1.847)	1.184	0.238
TREM2	0.094 (-0.208 – 0.396)	0.614	0.539	0.237 (-0.201 – 0.674)	1.065	0.288	-0.697 (-1.803 – 0.408)	-1.244	0.215
YKL-40	0.038 (-0.213 – 0.29)	0.301	0.764	0.083 (-0.322 – 0.488)	0.405	0.686	0.689 (-0.577 – 1.954)	1.073	0.285
VEGF-A	-0.082 (-0.413 – 0.248)	-0.492	0.623	0.08 (-0.461 – 0.622)	0.292	0.770	0.467 (-1.179 – 2.113)	0.559	0.577
VEGF-B	0.009 (-0.285 – 0.303)	0.060	0.952	0.036 (-0.344 – 0.417)	0.188	0.851	0.586 (-0.758 – 1.931)	0.860	0.391
VEGF-C	0.078 (-0.204 – 0.361)	0.544	0.587	-0.033 (-0.452 – 0.386)	-0.155	0.877	-0.239 (-1.289 – 0.81)	-0.449	0.654
VEGF-D	-0.08 (-0.286 – 0.125)	-0.769	0.443	-0.041 (-0.415 – 0.333)	-0.217	0.828	0.535 (-0.526 – 1.595)	0.993	0.322
PGF	-0.036 (-0.244 – 0.171)	-0.343	0.732	0.124 (-0.289 – 0.538)	0.592	0.554	0.346 (-0.689 – 1.382)	0.659	0.510
NRGN	-0.079 (-0.281 – 0.124)	-0.765	0.445	0.28 (-0.134 – 0.693)	1.332	0.184	1.039 (-0.062 – 2.14)	1.860	0.064
NPTX2	0.112 (-0.126 – 0.351)	0.928	0.354	0.206 (-0.042 – 0.454)	1.638	0.102	1.202 (-0.01 – 2.415)	1.955	0.052
SV2A	0.018 (-0.226 – 0.262)	0.143	0.886	-0.203 (-0.491 – 0.085)	-1.389	0.166	-0.147 (-1.32 – 1.026)	-0.247	0.805
SYT1	0.087 (-0.087 – 0.261)	0.986	0.325	0.07 (-0.224 – 0.365)	0.471	0.638	0.314 (-0.445 – 1.073)	0.816	0.416
14-3-3 ζ/δ	0.061 (-0.15 – 0.272)	0.569	0.570	0.05 (-0.416 – 0.516)	0.209	0.834	-0.039 (-1.299 – 1.221)	-0.061	0.951
NfL	-0.027 (-0.245 – 0.191)	-0.245	0.806	-0.154 (-0.602 – 0.294)	-0.677	0.499	-0.576 (-1.645 – 0.493)	-1.062	0.289
NGF	0.091 (-0.072 – 0.255)	1.099	0.273	0.32 (0.023 – 0.616)	2.124	**0.035**	0.351 (-0.548 – 1.25)	0.770	0.442

* $p_{FDR} < 0.05$; ** $p_{FDR} < 0.01$; *** $p_{FDR} < 0.001$

Supplementary table 24. Main cross-sectional effects of CSF proteins on Trailmaking test A controlling for temporal meta-ROI tau.

Cognitive resilience (CR); main cross-sectional effects on TMTA across all diagnostic groups controlling for temporal meta-ROI tau									
	A+ CU			A+ MCI			A+ AD		
Variable	Std β coefficient (CI)	t	p	Std β coefficient (CI)	t	p	Std β coefficient (CI)	t	p
GFAP	0.084 (-0.434 – 0.603)	0.321	0.749	0.132 (-0.848 – 1.112)	0.266	0.790	0.613 (-1.296 – 2.522)	0.634	0.527
GRN	-0.317 (-1.177 – 0.543)	-0.726	0.468	-0.483 (-1.67 – 0.705)	-0.802	0.424	0.221 (-3.012 – 3.454)	0.135	0.893
ICAM-1	0.109 (-0.511 – 0.728)	0.345	0.730	0.074 (-0.619 – 0.767)	0.210	0.834	0.088 (-2.276 – 2.453)	0.074	0.941
IL-15	-0.048 (-0.594 – 0.498)	-0.173	0.863	-0.012 (-1.139 – 1.115)	-0.021	0.984	-0.681 (-2.464 – 1.102)	-0.754	0.452
TREM2	0.073 (-0.593 – 0.74)	0.217	0.829	-0.219 (-1.329 – 0.891)	-0.389	0.698	-0.642 (-2.658 – 1.375)	-0.628	0.531
YKL-40	0.115 (-0.468 – 0.699)	0.390	0.697	0.222 (-0.766 – 1.211)	0.444	0.658	-0.173 (-2.343 – 1.998)	-0.157	0.875
VEGF-A	0.057 (-0.745 – 0.858)	0.140	0.889	0.14 (-1.213 – 1.493)	0.205	0.838	1.784 (-0.869 – 4.436)	1.327	0.186
VEGF-B	-0.306 (-0.982 – 0.369)	-0.894	0.373	0.156 (-0.772 – 1.084)	0.332	0.741	-0.325 (-2.103 – 1.453)	-0.361	0.719
VEGF-C	0.175 (-0.456 – 0.807)	0.547	0.585	0.002 (-1.037 – 1.041)	0.004	0.997	0.251 (-1.446 – 1.949)	0.292	0.770
VEGF-D	-0.14 (-0.657 – 0.377)	-0.536	0.593	0.311 (-0.569 – 1.19)	0.697	0.487	-0.38 (-2.148 – 1.388)	-0.424	0.672
PGF	0.141 (-0.367 – 0.649)	0.547	0.585	-0.295 (-1.269 – 0.679)	-0.597	0.551	0.031 (-1.675 – 1.737)	0.036	0.971
NRGN	-0.227 (-0.738 – 0.284)	-0.875	0.383	0.151 (-0.842 – 1.143)	0.299	0.765	0.594 (-1.187 – 2.376)	0.659	0.511
NPTX2	0.267 (-0.327 – 0.861)	0.886	0.377	0.244 (-0.404 – 0.891)	0.743	0.458	0.873 (-1.05 – 2.796)	0.896	0.372
SV2A	-0.272 (-0.846 – 0.302)	-0.936	0.350	0.126 (-0.642 – 0.894)	0.324	0.746	1.278 (-0.611 – 3.168)	1.336	0.183
SYT1	0.159 (-0.34 – 0.658)	0.629	0.530	-0.091 (-0.913 – 0.732)	-0.218	0.828	0.924 (-0.357 – 2.205)	1.424	0.156
14-3-3 ζ/δ	-0.246 (-0.816 – 0.324)	-0.852	0.396	0.254 (-0.987 – 1.496)	0.404	0.686	0.874 (-1.239 – 2.986)	0.817	0.415
NfL	0.032 (-0.528 – 0.591)	0.112	0.911	0.08 (-1.042 – 1.203)	0.141	0.888	-0.436 (-2.183 – 1.312)	-0.492	0.623
NGF	0.126 (-0.266 – 0.517)	0.633	0.528	-0.302 (-1.036 – 0.433)	-0.81	0.419	-0.663 (-2.115 – 0.789)	-0.902	0.369

* $p_{FDR} < 0.05$; ** $p_{FDR} < 0.01$; *** $p_{FDR} < 0.001$

Supplementary table 25. Interactive effects of CSF proteins with temporal meta-ROI tau on longitudinal global cognition in the BR sample.

	A+ CU (mPACC5)			A+ MCI (MMSE)			A+ AD (MMSE)		
Variable	Std β coefficient (CI)	t	p	Std β coefficient (CI)	t	p	Std β coefficient (CI)	t	p
GFAP	-0.074 (-0.110 – -0.038)	-4,029	**<0.001****	0.053 (-0.011 – 0.117)	1,643	0,104	-0.052 (-0.101 – -0.003)	-2,105	**0,038**
GRN	-0.044 (-0.101 – 0.013)	-1,536	0,127	0.023 (-0.034 – 0.080)	0,790	0,432	-0.018 (-0.061 – 0.024)	-0,866	0,388
ICAM-1	-0.056 (-0.104 – -0.007)	-2,269	**0,024**	0.010 (-0.055 – 0.075)	0,310	0,758	-0.003 (-0.045 – 0.038)	-0,160	0,873
IL-15	-0.084 (-0.137 – -0.032)	-3,181	**0,002***	0.043 (-0.019 – 0.104)	1,379	0,171	-0.012 (-0.054 – 0.031)	-0,538	0,592
TREM2	-0.039 (-0.084 – 0.007)	-1,692	0,093	0.030 (-0.021 – 0.081)	1,175	0,243	-0.020 (-0.059 – 0.020)	-1,001	0,319
YKL-40	0.018 (-0.035 – 0.072)	0,675	0,501	0.010 (-0.054 – 0.073)	0,307	0,760	0.003 (-0.054 – 0.060)	0,108	0,914
VEGF-A	-0.101 (-0.156 – -0.047)	-3,677	**<0.001****	0.018 (-0.034 – 0.070)	0,700	0,485	-0.019 (-0.055 – 0.017)	-1,057	0,293
VEGF-B	-0.087 (-0.143 – -0.03)	-3,011	**0,003***	0.014 (-0.057 – 0.086)	0,399	0,691	-0.01 (-0.041 – 0.0220)	-0,609	0,544
VEGF-C	-0.038 (-0.096 – 0.02)	-1,295	0,198	0.024 (-0.031 – 0.079)	0,882	0,380	-0.008 (-0.042 – 0.026)	-0,489	0,626
VEGF-D	-0.102 (-0.146 – -0.058)	-4,618	**<0.001*****	0.043 (-0.005 – 0.091)	1,757	0,082	-0.005 (-0.042 – 0.031)	-0,277	0,782
PGF	-0.078 (-0.131 – -0.025)	-2,878	**0,004***	0.011 (-0.043 – 0.064)	0,402	0,689	-0.040 (-0.088 – 0.009)	-1,625	0,108
NRGN	-0.099 (-0.154 – -0.043)	-3,528	**0,001****	0.043 (-0.028 – 0.115)	1,211	0,229	0.017 (-0.013 – 0.048)	1,146	0,256
NPTX2	-0.019 (-0.078 – 0.04)	-0,646	0,520	0.064 (0.011 – 0.116)	2,401	**0,019**	0.020 (-0.016 – 0.056)	1,095	0,276
SV2A	-0.052 (-0.130 – 0.026)	-1,336	0,185	0.014 (-0.075 – 0.102)	0,306	0,761	0.002 (-0.043 – 0.046)	0,073	0,942
SYT1	-0.073 (-0.145 – -0.001)	-2,008	**0,047**	0.043 (-0.041 – 0.127)	1,019	0,311	0.010 (-0.034 – 0.055)	0,464	0,643
14-3-3 ζ/δ	-0.092 (-0.158 – -0.026)	-2,744	**0,007***	0.039 (-0.057 – 0.134)	0,804	0,424	0.021 (-0.039 – 0.080)	0,692	0,491
NfL	-0.091 (-0.132 – -0.05)	-4,359	**<0.001*****	0.008 (-0.058 – 0.075)	0,244	0,808	-0.029 (-0.084 – 0.026)	-1,055	0,294
NGF	0.100 (0.067 – 0.133)	6,004	**<0.001*****	0.008 (-0.061 – 0.078)	0,230	0,819	0.011 (-0.035 – 0.058)	0,488	0,627

* $p_{FDR} < 0.05$; ** $p_{FDR} < 0.01$; *** $p_{FDR} < 0.001$
